# Molecular profiling of high-level athlete skeletal muscle after acute endurance or resistance exercise – A systems biology approach

**DOI:** 10.1016/j.molmet.2023.101857

**Published:** 2023-12-21

**Authors:** Stefan M. Reitzner, Eric B. Emanuelsson, Muhammad Arif, Bogumil Kaczkowski, Andrew TJ. Kwon, Adil Mardinoglu, Erik Arner, Mark A. Chapman, Carl Johan Sundberg

**Affiliations:** 1 Department Physiology & Pharmacology, Karolinska Institutet, Solnavägen 9, 171 77 Stockholm, Sweden; 2 Department Women's and Children's Health, Karolinska Institutet, Solnavägen 9, 171 77 Stockholm, Sweden; 3 Science for Life Laboratory, KTH - Royal Institute of Technology, Tomtebodavägen 23, 171 65 Stockholm, Sweden; 4 Center for Integrative Medical Sciences, RIKEN Yokohama, 1 Chome-7-22 Suehirocho, Tsurumi Ward, Yokohama, Kanagawa 230-0045, Japan; 5 Centre for Host-Microbiome Interactions, Faculty of Dentistry, Oral & Craniofacial Sciences, King's College London, Guy's Hospital, Great Maze Pond, London, SE1 1UL, United Kingdom; 6 Graduate School of Integrated Sciences for Life, Hiroshima University, 1 Chome-3-3-2 Kagamiyama, Higashi-Hiroshima, Hiroshima 739-0046, Japan; 7 Department Physiology & Pharmacology, Department Women's and Children's Health, Karolinska Institutet, Solnavägen 9, 171 77 Stockholm, Sweden; 8 Department of Integrated Engineering, University of San Diego, 5998 Alcalà Park, San Diego, CA 92110, USA; 9 Department of Learning, Informatics, Management and Ethics, Karolinska Institutet, Tomtebodavägen 18A, 171 65 Solna, Sweden; 10 Department of Laboratory Medicine, Karolinska Institutet, Alfred Nobels Allé 8, 141 52 Huddinge, Sweden

**Keywords:** Molecular exercise effects, Metabolomics, Multi-omics, Human, Systems biology, Athletes

## Abstract

**Objective:**

Long-term high-level exercise training leads to improvements in physical performance and multi-tissue adaptation following changes in molecular pathways. While skeletal muscle baseline differences between exercise-trained and untrained individuals have been previously investigated, it remains unclear how training history influences human multi-omics responses to acute exercise.

**Methods:**

We recruited and extensively characterized 24 individuals categorized as endurance athletes with >15 years of training history, strength athletes or control subjects. Timeseries skeletal muscle biopsies were taken from *M. vastus* lateralis at three time-points after endurance or resistance exercise was performed and multi-omics molecular analysis performed.

**Results:**

Our analyses revealed distinct activation differences of molecular processes such as fatty- and amino acid metabolism and transcription factors such as HIF1A and the MYF-family. We show that endurance athletes have an increased abundance of carnitine-derivates while strength athletes increase specific phospholipid metabolites compared to control subjects. Additionally, for the first time, we show the metabolite sorbitol to be substantially increased with acute exercise. On transcriptional level, we show that acute resistance exercise stimulates more gene expression than acute endurance exercise. This follows a specific pattern, with endurance athletes uniquely down-regulating pathways related to mitochondria, translation and ribosomes. Finally, both forms of exercise training specialize in diverging transcriptional directions, differentiating themselves from the transcriptome of the untrained control group.

**Conclusions:**

We identify a “transcriptional specialization effect” by transcriptional narrowing and intensification, and molecular specialization effects on metabolomic level Additionally, we performed multi-omics network and cluster analysis, providing a novel resource of skeletal muscle transcriptomic and metabolomic profiling in highly trained and untrained individuals.

## Highlights

• Carnitine metabolites are more abundant in long-term endurance trained athletes.
• Sorbitol is a major regulated metabolite following all forms of acute exercise.
• Athletes specialize by amplification of an increasingly focused molecular response.
• Key clusters of acute exercise focus on TCA cycle and fatty acid metabolism.
• 5 analytes can predict up to 68 % of physical performance measures.

## 1 Introduction

High-level physical training results in exercise modality-specific adaptations in skeletal muscle that are partly due to transient as well as accumulating changes in gene expression, protein levels and modifications as well as in metabolite levels [[1], [2], [3]]. Following each exercise bout, such changes are influenced by an interaction between signaling molecules and the transcriptional machinery, over time contributing to the gradual remodeling of skeletal muscle [3]. The structural and biochemical remodeling that has accrued in trained skeletal muscle, in turn, influences its transcriptional and metabolic response to acute exercise bouts [4,5]. Previous investigations of skeletal muscle gene expression following acute exercise bouts or exercise training programs [4,[6], [7], [8]] have mostly been limited either by exercise intervention duration (weeks to months) or training status of the population studied (untrained to recreationally active). Other investigations of acute exercise effects have focused on blood only [9]. Accordingly, to overcome such limitations, we recruited two groups of long-term trained (15+ years) endurance and resistance athletes and one age-matched untrained control group. All participants performed acute bouts of endurance and resistance exercise in a cross-over design before and after which biopsies were obtained from skeletal muscle on which multi-omics analyses were conducted.

The aim was to identify key molecular pathways connected to physical performance capabilities, comparing group differences in training history and modality of acute exercise to better understand the molecular trajectories following acute exercise. During subject characterization, several performance parameters were measured. The association between such markers and molecular level pathways and metabolites can be of great value for the identification of processes that drive skeletal muscle performance and health. Increased understanding of such molecular characteristics can be clinically relevant for the development of strategies to prevent, treat and assess the status of diseases like diabetes, metabolic syndrome, cardiovascular disease, cancer or enabling beneficial exercise-induced effects when exercise is not an option. Furthermore, an increased understanding of the biology in long-term trained athletes could provide valuable knowledge for training protocol optimization by refocusing on training modalities that trigger key performance-relevant adaptations such as introducing endurance and resistance elements in strength and endurance athletes, respectively. While efforts have previously been made to understand and identify such processes in different tissues or with short duration exercise interventions [9], no study to date has systematically investigated the influence of long-term high-level training on the collective transcriptome, metabolome and transcription factor (TF) motifs response to different forms of acute exercise in human skeletal muscle.

Results from our comprehensive characterization of the transcriptomic, metabolomic and regulatory response to acute exercise show extensive differences based on training history, amongst others due to sports-specific specializations in energy metabolic pathways. The results reveal distinct molecular differences between acute endurance and resistance exercise and, by using correlation analysis identify key metabolic processes that could act as key drivers to performance output such as carnitine and amino acid metabolisms. Taken together, we deliver for the first time, a multi-omics characterization of skeletal muscle from highly trained individuals. Additionally, we provide a valuable, easy to use public resource (https://bit.ly/iNetModels_CrossEX (Multi-omics network → Study-specific Networks → crossEX (Reitzner et al., 2024)) for future investigations of the molecular effects of acute exercise and long-term adaptation, highlighting the potential of a multi-omics profiling approach.

## 2 Methods

### Ethical approval

Before the interventions, all subjects were informed about the study outline, familiarized with the experimental procedures and potential complications and written and verbal consent was obtained. The study was approved by the Stockholm regional ethics board (Dnr: 2016/590-31) under observance of the declaration of Helsinki.

### 2.1 Participant recruitment and inclusion

Twenty-four healthy, non-smoking men aged 33–52 were recruited after filling in a questionnaire to assess their training history and health status and a selection process including peak oxygen uptake test ($\dot{V}$O_2_peak test) and maximum knee torque test measured by unilateral isokinetic Biodex test (Biodex System 4, Biodex Medical Systems, Shirley (NY), USA) to fit into one of three groups: resistance trained (SG), endurance trained (EG) and control (CG). $\dot{V}$O_2_peak test was performed on a stationary bike and initial resistance individually set depending on expected subject performance. After 5 min of warm-up cycling, resistance was increased incrementally between 16.6 and 26.6 W⋅min^−1^ until exhaustion. All subjects reaching a respiratory exchange ratio (RER) above 1.05. Inclusion into the EG required a $\dot{V}$O2peak above the 90th percentile of the subject's age group [10]. Additionally, knee extension 1 repetition maximum (1RM) was measured and controlled using the Borg RPE scale [11]. Inclusion into the SG required a peak torque of at least two standard deviations above the mean of the control group (Table 1). The resistance and endurance trained athletes were required to have either a resistance- or an endurance-based training history at high level of at least 15 years respectively. These groups were clearly separated by both, $\dot{V}$O2peak and maximum knee torque (Table 1). Thresholds for $\dot{V}$O2peak and peak torque were selected to be identical to our previously published cross-sectional study to ensure maximal comparability [12].

**Table 1:** Basic characterization of research subjects.

Group	Control (CG)	Endurance (EG)	Strength (SG)
n	8	8	8
Age (yrs)	44 ± 6	42 ± 5	39 ± 6
Height (cm)	181 ± 6	182 ± 4	180 ± 5
Weight (kg)	76 ± 8	73 ± 4	92 ± 12 ∗
BMI (kg/m^2^)	23.1 ± 2.4	22.3 ± 0.5	28.3 ± 2.9 ∗
Leg strength (Nm)	180.6 ± 30.8	198.3 ± 25.5	286.4 ± 28.1 ∗
Leg strength (1RM; kg)	39.35 ± 7.02	51.13 ± 4.98 ^**#**^	75.45 ± 8.55 ∗
$\dot{V}$O2 peak (ml⋅kg^−1^⋅min^−1^)	36.2 ± 4.4	69 ± 7.2 ∗	40.2 ± 6.9
$\dot{V}$E/$\dot{V}$CO_2_ slope	35.8 ± 5.9 ∗	28.9 ± 2.8	30.2 ± 1.6
CS activity (nmol⋅min^−1^⋅mg protein^−1^)	5.0 ± 0.5	10.1 ± 1.5 ∗	4.2 ± 0.7
Proportion type 1 fibers (%)	44.6 ± 4.4	61.4 ± 3.9 ∗	43.0 ± 3.2
Proportion type 2 fibers (%)	55.4 ± 4.4	38.6 ± 3.9 ∗	57 ± 3.2
Type 1 CSA (μm^2^)	4841.7 ± 561.2 ∗	6048.3 ± 257.8	6660.6 ± 522.3
Type 2 CSA (μm^2^)	5369.5 ± 860.3	6632 ± 665.2 ^**#**^	9184 ± 415.2 ∗
Nuclei/type 1 fiber	2.6 ± 0.6	3.4 ± 0.5 ^**#**^	4.4 ± 0.6 ∗
Nuclei/type 2 fiber	2.9 ± 0.9	2.6 ± 0.8	4.5 ± 0.9 ∗

Values are group mean ± SD. (∗: p < 0.05 compared to both the other groups; ^#^: p < 0.05 compared to CG. $\dot{V}$E = minute volume, $\dot{V}$CO_2_ = volume of exhaled carbon dioxide, CS = citrate synthase, CSA = cross-sectional area).

### 2.2 Study design

**Figure 1:**
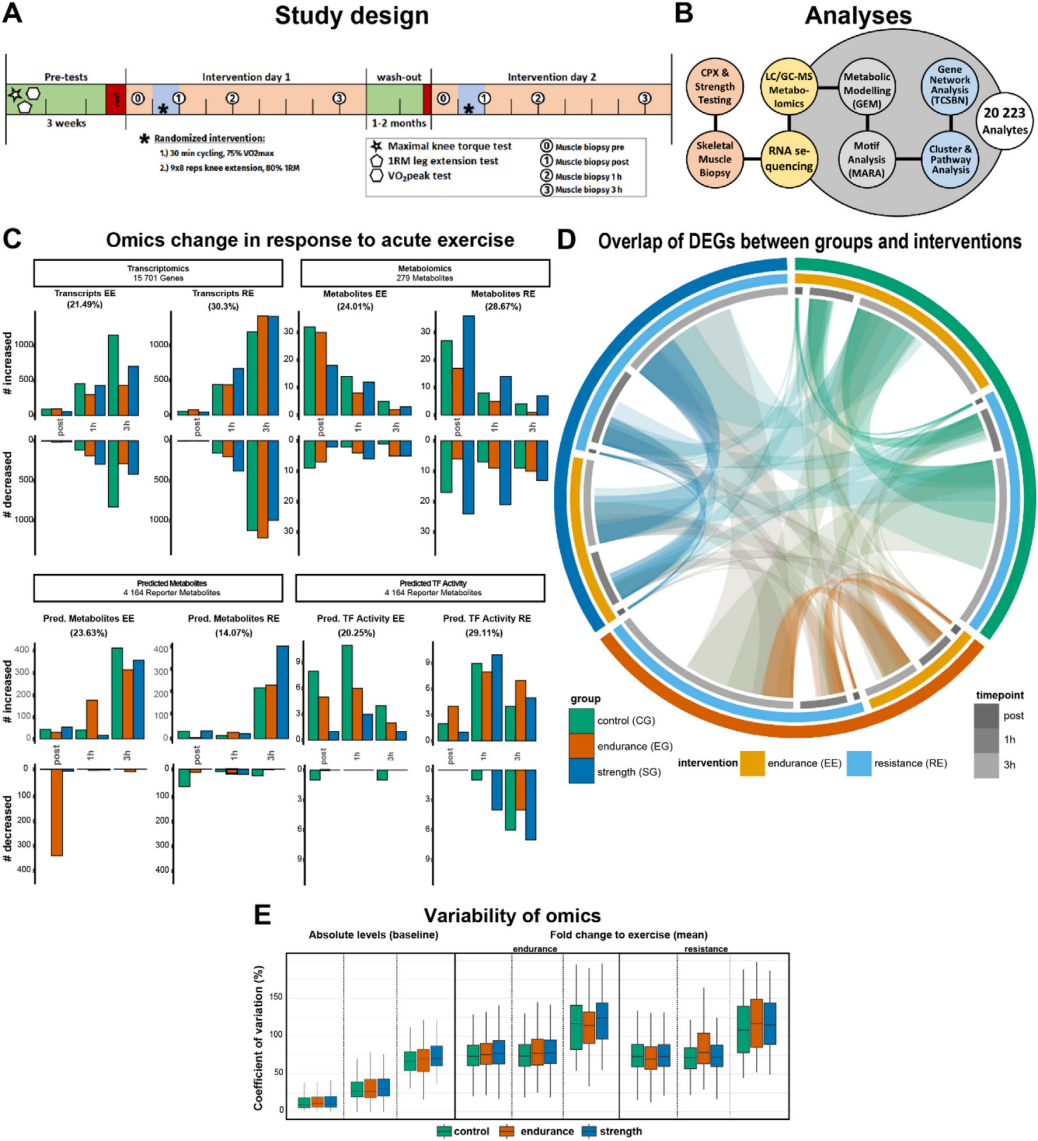
**Study Design, Molecular Response to Exercise and overlap of DEGs**, (A) Overview over the study design. Two bouts of endurance (EE) or resistance exercise (RE) were performed in a randomized cross-over design and skeletal muscle biopsies taken at 4 timepoints. (B) Cardiopulmonary (CPX) and strength testing and analyses performed. (C) Multi-omics response to acute exercise by subject group with the percentage of total significantly affected by EE or RE. (D) Overlap of differentially expressed genes compared to the respective pre timepoint. Widths of arches and segment lengths are proportional to the number of DEGs. (E) Within subject group coefficient of variation of analytes (transcripts, directly measured metabolites and transcription factor motifs) at baseline and in response to acute endurance or resistance exercise (mean of time course).

All subjects completed both a single bout of EE and RE in randomized order with at least one month of wash-out in between (Figure 1A). Food intake was controlled for by a standardized breakfast and the instruction to eat the same dinner on the day before each of the intervention days. After a standardized breakfast 3 h prior to the first biopsy timepoint, subjects arrived between 8:00 and 9:00 in the morning at the Division for Clinical Physiology research unit at the Karolinska Hospital in Huddinge, Sweden. Subjects were then randomized to either an acute endurance or acute resistance exercise session. After a resting skeletal muscle biopsy was taken using the Bergström needle technique with suction from *M. vastus lateralis*, subjects performed 30 min of acute exercise. Subjects were pushed to their maximum performance in each session. For acute endurance exercise, subjects were required to cycle at 75 % of their individual W_max_ for 30 min. For acute resistance exercise, subjects were required to complete 9 sets of 8 repetitions of knee extension (set length 40 s; set break, 150 s) exercise at 80 % of their individual one repetition maximum. Following the conclusion of the acute exercise, three additional skeletal muscle biopsies were taken from *M. vastus lateralis* immediately following acute exercise and 1 h and 3 h after the end of the acute exercise. Between 4 and 8 weeks after the first session, all subjects returned to perform the form of exercise they have not been randomized for the first occasion and skeletal muscle biopsies were again collected at a total of four timepoints.

### 2.3 Skeletal muscle tissue biopsies

Skeletal muscle biopsies were taken alternating from left and right *M. vastus* lateralis before and directly after and at 1 h and 3 h after one single bout of exercise using the Bergström needle technique [13]. To prevent possible dominant/non-dominant leg effects, the first muscle biopsy was taken from either the dominant or the non-dominant leg by randomization. The skeletal muscle biopsies were then snap-frozen using 2-methylbutane as a secondary coolant.

### 2.4 Baseline tissue characterization

#### 2.4.1 Enzyme extraction and enzymatic assays

Enzymes were extracted using a phosphate-based buffer (50 mM KH2PO4, 1 mM EDTA, 0.05 % Triton X-100) and quantified by Bradford assay (Bio-Rad #5000006, Bio-Rad, Hercules, CA, USA). Citrate synthase activity was measured spectrophotometrically as previously described [14].

### 2.5 Immunohistochemical fiber typing

Skeletal muscle samples were cut on a cryostat (CryoStar NX70, Thermo Scientific, Waltham, MA, USA) at 7 μm and immunohistochemically stained for Type I (DSHB, Cat# BA-F8, RRID:AB_10572253) and Type II (DSHB, Cat# SC-71, RRID:AB_2147165) fibers and Laminin (Sigma-Aldrich, Cat# L9393, RRID:AB_477163). Compatible fluorescent antibodies were used for detection with the Olympus IX-73 wide-field fluorescence microscope (Olympus Corp., Shinjuku, Tokyo, Japan). Analysis of immunohistochemically stained cross-sectional cuts was performed blinded using ImageJ.

### 2.6 RNA sequencing

RNA extraction from skeletal muscle tissue was performed using the phenol based TRIzol method (Invitrogen #15596018, Thermo Fisher Scientific, Waltham, MA, USA), quantified and quality checked using the 2100 Bioanalyzer System (Agilent Technologies, Santa Clara, CA, USA). Libraries were prepared by poly-A selection (TruSeq mRNA, Illumina, San Diego, CA, USA) and multiplexed at the National Genomics Infrastructure Sweden. Clustering was done by ‘cBot’ and samples were sequenced on NovaSeq6000 (NovaSeq Control Software 1.6.0/RTA v3.4.4) with two lanes of a 2x151 setup ‘NovaSeqXp’ workflow in ‘S4’ mode flow cell. The Bcl to FastQ conversion was performed using bcl2fastq_v2.20.0.422 from the CASAVA software suite. The quality scale used is Sanger/phred33/Illumina 1.8+. QC and processing were performed using the nfcore/rnaseq analysis pipeline publicly available at github (https://github.com/nf-core/rnaseq). The RNA-sequencing data has been deposited at the European Genome-phenome Archive (EGA) which is hosted at the EBI and the CRG, under accession number EGA: EGAS00001006139.

### 2.7 Semi-targeted metabolomics by gas- and liquid chromatography (GC/LC)-MS

Metabolic profiling by GC-MS and LC-MS was performed at the Swedish Metabolomics Center in Umeå, Sweden. For more details see supplemental methods. Metabolomics data is available as supplement to this paper.

### 2.8 Quantification and statistical analysis

Statistical analyses were performed with R version 3.6.0. For baseline analyses, ANOVA and t-test were used. Gene expression was measured by differential gene expression determined by edgeR package analysis [15]. Analyses were also performed with subject groups or acute pooled to provide results for a background-independent general training effect. Repeated testing corrections were performed using the Benjamini-Hochberg method [16]. A searchable excel-format differential gene expression analysis results data table is available as Supplement B to this paper. Using the data from differential gene expression analysis, gene set enrichment was determined by fGSEA based on log(FC)-sorting using the GO collections “cellular component”, “molecular function” and “biological process”. Gene and metabolite clustering was performed using k-means unsupervised clustering.

Area under der curve (AUC) for each gene was calculated using trapezoid integration and used for correlation analysis with baseline physiological performance markers $\dot{V}$O_2_peak and peak torque using Pearson's correlation coefficient. Modelling of top genes explaining $\dot{V}$O2peak and peak torque was performed with linear modelling.

Genome-scale Metabolic Modelling (GEM) was performed using RNA sequencing results. Using GEMs, metabolites and metabolic pathways can be inferred by modelling metabolome information onto RNA sequencing results. Generic human model, HMR version 2.00 was used to generate gene-metabolite networks as previously described [17].

Regulatory motif analysis was performed using a motif activity response analysis (MARA) algorithm [18] and the FANTOM5 database of regulatory annotation [[19], [20]]. Of the 192 available motifs from the FANTOM5 annotation, 79 were left after running the MARA algorithm. These transcription factors motifs were assessed for their member genes that drive the temporal shape of the response to acute exercise. Only DEGs that were considered differentially regulated in the respective comparison in the differential gene expression analysis were considered for correlation to the motif using Pearson's correlation coefficient. Verification of gene-gene interactions within regulatory motifs was performed using the tissue and cancer specific biological networks (TCSBN) database [21]. The multi-omics network was generated by using the same pipeline/method as described in iNetModels [[21], [22]].

Alpha was set to 0.01, unless stated otherwise, error bars represent standard error of the mean.

Further information and request for resources and reagent should be directed to and will be fulfilled by the lead contact, Stefan Markus Reitzner (stefan.reitzner@ki.se).

## 3 Results

### 3.1 Subject characterization and analyses performed

Highly characterized healthy male subjects (n = 24) between 33 and 52 were recruited into three groups based on self-reported training history and a series of physiological performance tests ($\dot{V}$O2peak, 1 repetition maximum and peak torque knee extension, Table 1): long-term high-level trained endurance (EG) and strength (SG) athletes and age-matched untrained healthy participants (CG). Inclusion criteria were based on leg strength, $\dot{V}$O_2_peak test and training history and identical to our previously published cross-sectional study [12]. The research subjects underwent a series of skeletal muscle biopsies before and after two acute exercise interventions separated by 1–2 months (Figure 1A). Subsequently, RNA sequencing (transcriptome), liquid and gas chromatography-mass spectrometry (metabolome, lipidome), and *in-silico* genome scale metabolic modelling (GEM) [17] and TF motif activity response analysis (MARA) [23] were performed (Figure 1B). In addition to 11 physiological and histological parameters (see Table 1), the final dataset included a total of 20223 analytes, composed of: 15701 genes, 279 metabolites, 4164 reporter metabolites – markers of in-silico inferred metabolic flux, and 79 TF motifs (Figure 1C).

### 3.2 Baseline physiological, biochemical and histological differences

By design, leg strength was significantly higher in the strength group (SG) compared to both control group (CG) and endurance group (EG; Table 1) without any overlap. $\dot{V}$O2peak in EG was significantly higher than both other groups, and SG leg strength significantly higher than both EG and CG. Similarly, skeletal muscle citrate synthase (CS) activity was significantly higher in EG than both other groups, a consequence of long-term endurance training [24]. Skeletal muscle type 1 fiber proportion was significantly higher in EG compared with both other groups (Table 1, Figure S1), a difference that has been observed before [[25], [26]]. Muscle fiber cross-sectional area (CSA) was significantly lower in CG for type 1 compared to both other groups. EG and SG had a similar type 1 CSA but a significantly larger type 2 CSA was observed in SG (Table 1, Figure S1).

### 3.3 Molecular inter- and intra-group differences at baseline

#### 3.3.1 Variability of omics

Descriptive statistical analyses on our dataset (Figure 1E,B, Figure S2B–E) demonstrated that the within-group coefficient of variation (CoV) of analytes at baseline was similar in all groups but varied depending on omics-level (Figure 1E left), demonstrating a consistent intra-group variability of omics across groups at baseline with no significant group difference. Previous publications have reported long-term exercise training to have a consolidating effect for individual molecular analytes [27] or in response to acute exercise [12], increasing similarity between athletes. Our results showed that intra-group analyte-wise variation was similar across groups regardless of training status (Figure 1E, Figure S2E), suggesting that the molecular adaptation of high-level athletes lies in the fine-tuning of pathways and individual genes and metabolites rather than in the overall omics dimension. When comparing intra-individual gene expression between the two pre timepoints, small differences were found, with a mean fold difference close to zero, and only 3.12–3.96 % of all transcripts displaying a fold change of 0.5 or higher (Figure S2B). These results provide an interesting benchmark of inherent, day-to-day shift of individual baseline gene expression. These can be the product of slight differences in daily conditions regarding nutrition, sleep, immune system status or other factors. While we are confident that we tightly controlled for some of the above-mentioned factors insofar possible, remaining variation can be a product of random or environmental factors such as seasonality, which up to 10 % of all genes in skeletal muscle might be influenced by [28]. However, with the average distance between intervention days being 55 days this should not be a major factor. Taken together, our data indicates a low level of baseline gene expression variability.

#### 3.3.2 Gene expression and metabolomic group differences

Comparing gene expression at baseline, the results showed smaller differences between CG and SG (57 DEGs) than between the EG and both other groups (1363 DEGs vs CG and 919 DEGs vs SG; Supplement A, Table S1), similar to what has previously been shown by us [12]. Using gene set enrichment analysis (GSEA), we show that these DEGs are associated with mitochondrial pathways enriched in the endurance group, while differences between CG and SG are mainly attributed to immune system and structural terms (Figure S2D).

On metabolomic level, SG showed to have high inter-individual diversity of metabolite levels of sorbitol, gluconic acid and erythritol (Figure S2A), all previously reported to play a major role in skeletal muscle glucose metabolism in rodents [[29], [30], [31]]. We found metabolic baseline differences between groups (see Supplement A, Table S2 and Figure S2C). Specifically, carnitines were more abundant in the EG compared to both other groups, representing about 20 % of the significantly different metabolites. Carnitines act as intracellular chaperone molecules for beta-oxidation via the carnitine-palmitoyltransferase system which have a rate-limiting role [[32], [33]] for the transport of fatty acids across the mitochondrial membranes. They have been reported to be elevated in circulating blood of high-level trail runners [34]. High concentrations of different length fatty acids bound to their carnitine transporters, such as propryonyl- or butyrylcarnitine can be interpreted both as a higher potential for beta-oxidative capacity and higher levels of ongoing beta-oxidation at rest [35]. Such an adaptation is of great benefit for endurance exercise, but generally of less relevance to resistance exercise. About 30 % of all metabolites that were significantly more abundant in SG than in CG were the phospholipid groups of phosphatidylcholines (PC) and phosphatidylethanolamines (PE), of which 75 % were unsaturated and only 25 % saturated, which have previously been described as relevant for membrane fluidity [36], insulin receptor- and membrane protein dynamics [[37], [38]] and mitochondrial structure [39] (Figure S2C). The positive effect of RE on insulin sensitivity and membrane fluidity has previously been demonstrated [[40], [41]], and has been shown to be a consequence of a more flexible recruitment of GLUT4 into the plasma membrane [42], thus aiding glucose uptake. Together, both these metabolic PC-PE and carnitine profiles found in SG and EG, are consistent with the respective functional requirements of high-level endurance and resistance exercise.

### 3.4 Molecular response to acute exercise

**Figure 2:**
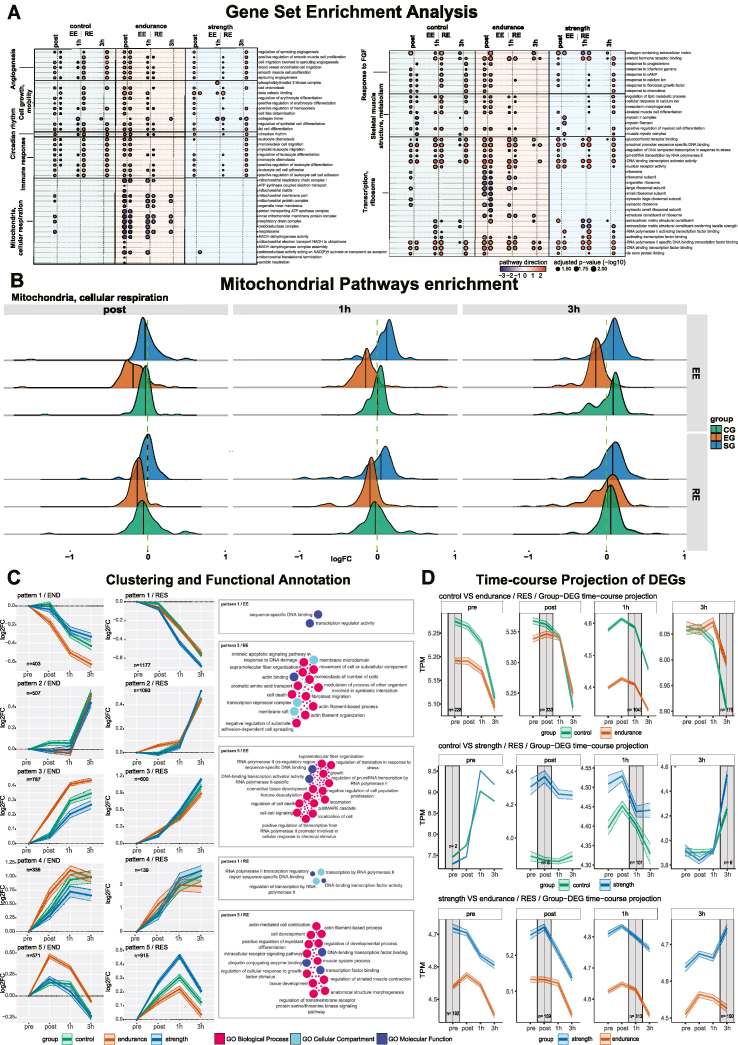
**Functional analysis of the transcriptomic response to acute endurance and resistance exercise**. (A) Gene set enrichment analysis (GSEA) of DEGs for acute endurance (EE) and resistance exercise (RE). Individual pathways are summarized by supergroups. (B) Ridge plot of mitochondrial and cellular respiration pathway molecular signatures from A. (C) Gene pattern identity from unsupervised clustering of genes of the endurance group performing EE (left) and the strength group performing RE (right) are used to compare the same sets of genes in all groups. Corresponding GSEA of patterns are shown on the far right in (C). Colors represent gene ontology collection of origin, labelled large dots are exercise-relevant pathways while small dots represent other pathways. (D) Projection (plotting of DEGs from one timepoint across the remaining timepoints; origin comparison timepoint with grey background) of DEGs comparing groups at individual timepoints to the remaining timepoints (mean ± confidence interval).

**Figure 3:**
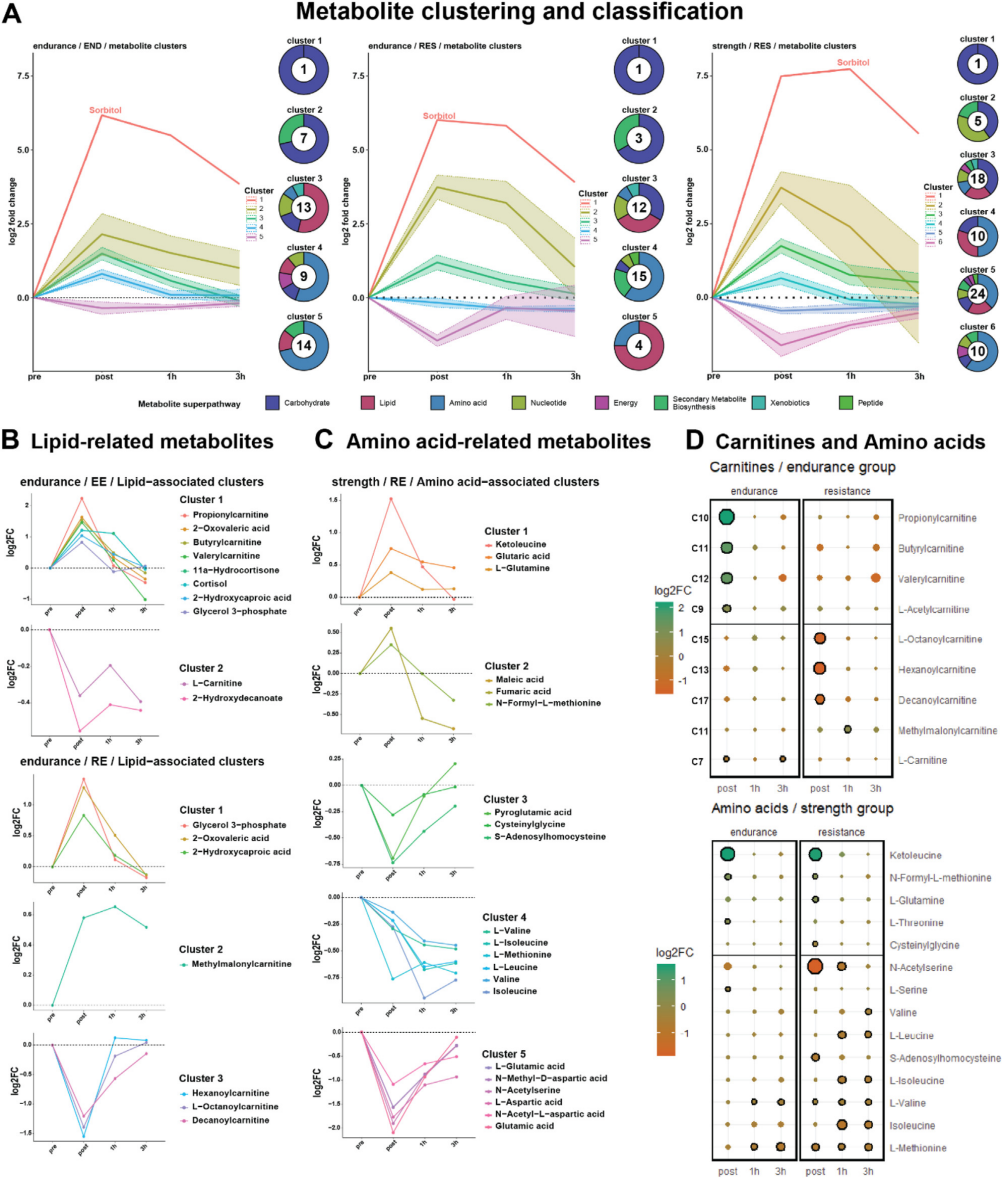
**Clustering and classification of metabolomics**. (A) Clustering (mean ± confidence interval) and classification of metabolites of endurance (EG) and strength group (SG) in response to acute endurance (EE) and resistance exercise (RE). Numbers in donuts represent the cluster size. (B) Clustering of lipid-associated metabolites in EG in response to EE and RE and (C) of amino acid-associated metabolites in SG in response to RE. (D) Analysis of carnitines in EG and amino acids in SG in response to acute exercise. Size and color of dots represent effect size and direction. Black circles represent statistical significance compared to pre.

The acute response to exercise has been suggested to follow a time-specific choreography, modulated by acute exercise modality and training background [[9], [43]]. To better understand this modulation, we compared trajectories of differentially expressed genes (DEGs). An overview can be found in Figure 3A. Together with the higher number of DEGs in response to RE (Figure 1C), this data shows a more robust activation of *M. vastus lateralis* gene expression in response to RE compared to EE. Exercise modality showed to have a bigger role for gene activation than training background as indicated by stronger connections between same type acute exercise than within subject group (Figure 1D, Figure S3B). The most unique set of differentially expressed genes was found at the 3h timepoint with as much as 84 % of genes in EG performing RE (Figure S3A). Interestingly, compared to the 3h timepoint, the earlier timepoints resulted in a more coordinated gene expression response as measured by numbers of highly enriched pathways identified by GSEA. Such a pattern might be explained by EG being highly capable of meeting acute energy requirements of RE, but exhibiting a chaotic, less coordinated response, similar to untrained individuals at later stages. In contrast, as a first indicator of a group difference in the response to acute exercise, CG and SG show a high degree of significantly enriched pathways at the 3h timepoint (Figure 2A), particularly in response to RE rather than EE.

#### 3.4.1 Functional annotation of differential gene expression

Further GSEA analysis showed angiogenesis-, immune system-, growth factor- and transcription-related pathways to be upregulated across all groups and both interventions (Figure 2A). However, pathways related to mitochondria/cellular respiration and transcription/ribosome were uniquely downregulated in EG in response to both EE and RE (Figure 2A), particularly at the post timepoint (Figure 2B). Here, the 5 out of 17 pathways of the mitochondria/cellular respiration class at the post timepoint in CG had an average normalized enrichment score (NES) of −1.83, while EG had −2.68 and the one pathway in SG -2.07. In both 1h and 3h timepoints, only EG had significant pathways which had an average NES of −1.89 and −1.81 respectively (Figure 2A). In the transcription and ribosome class at the post timepoint, due to the large proportion of down-regulated pathways, EG showed and average NES of 0.07, while CG and SG had 1.08 and 2.07 respectively. Mitochondria are a major target of skeletal muscle adaptation, particularly following endurance training, influencing their biogenesis [44], plasticity [45], increasing cristae density [45] and potential citrate cycle throughput as measured by CS activity [[46], [47], [48]], which was significantly higher in EG compared to both CG and SG (Table 1). As shown above, EG unlike both CG and SG has increased baseline enrichment of pathways contributing to mitochondrial biogenesis and building up their function (Figure S2D). The aforementioned downregulation (Figure 2A) might potentially represent this high mitochondrial capacity of EG, providing sufficient amounts of energy substrates during acute exercise, and immediate downregulation upon cessation of exercise beyond baseline levels due to a complete saturation with energy substrates. Since both the EG and the SG are highly specialized in their respective disciplines, it is likely that their acute exercise transcriptomic response is highly optimized. Considering this notion, we were interested in how the sets of genes stimulated in highly adapted athletes respond in both the groups naïve to that form of exercise, CG and SG being endurance training-naïve, and CG and EG being strength training-naïve. To do so, we performed unsupervised clustering of DEGs of EG performing EE, and SG performing RE. The same genes were then plotted as clusters for all groups, enabling a direct group comparison of each cluster of DEGs. Results show a more pronounced response in EG (pattern 1, 3, 5; Figure 2C) following EE, while the same was true in SG following RE (pattern 1, 5; Figure 2C). At the same time, all groups showed the same general response shape, thus similar transcriptomic programs being stimulated by acute exercise regardless of training history. This could suggest that the differences between highly trained and exercise-naïve individuals in the omics dimension lie in a more efficient and fine-tuned degree of gene expression changes, rather than a large re-arrangement of much of the transcriptome, notwithstanding possible large differences on the level of individual genes. Functional profiling of patterns 1, 3 and 5 in EE showed an association with regulation of transcription, structural, myofibrillar and translation related terms. RE patterns 1 and 5 were associated with regulation of transcription, developmental process, morphogenesis and growth. Overall, less distinct group differences were found following RE, which is in line with the above-mentioned similarity between CG and SG, suggesting a smaller transcriptional response to RE than to EE. We also performed this analysis based on clustering from CG, with some but less striking differences (Figure 3C; pattern 2 EE and 5 RE). Further comparison of group differences additionally supported this previously mentioned notion of a broad but fine-tuned acute gene regulation (Figure 2D, Figure S3D). For this, we used group-comparison DEGs from each timepoint and plotted summary-level expression across all timepoints (Figure 2D). Across the majority of comparisons in response to both RE and EE, gene trajectories are largely parallel, however staying clearly separated in most cases, especially comparing SG and EG (Figure 2D bottom panel, Figure S3C). Together, this suggests that the acute form of exercise dictates the pattern of the transcriptomic response, irrespective of training background, albeit with different amplitudes of the induced differential gene expression.

#### 3.4.2 Generation of a genome-scale metabolic network

To expand the perspective on the dynamics of metabolites in response to acute exercise, we used the transcriptomic data to generate a genome-scale metabolic network (GEM) by integration with an atlas of human metabolic equations and pathways [17]. Most notably, the generated GEM showed a large change in EE stimulated metabolic flux at the post timepoint in the EG but not in CG and SG. Immediately after EE, EG showed to have 362 significantly affected reporter metabolites, of which 332 were in the negative direction (Figure 1C). CG and SG showed 45 and 62 affected reporter metabolites respectively, mainly in the positive direction. Using KEGG, the EG reporter metabolites were largely mapped to energy-related metabolic processes such as TCA cycle and fatty acid metabolism (Figure S5A). These results further reinforce our GSEA-based findings of a large-scale downregulation of mitochondria and cellular respiration pathways immediately following EE in EG, but not in the CG and SG (Figure 2A,B). This drastic, immediate down-regulation following EE might indicate a tighter control over up- and downregulation of an acute exercise transcriptomic profile in EG. This is exemplified in an abrupt cessation of transcription of energy-pathway related genes immediately after the end of the acute exercise and a drop in ATP demand, compared to both CG and SG and can represent yet another important optimization of the molecular layout in EG.

#### 3.4.3 Metabolomics

After this first predictive perspective on metabolic flux, the abundance of 279 metabolites were directly measured by targeted LC/GC-MS. Following unsupervised *k*-means clustering of significantly changed metabolites [49], they were annotated by their metabolic superpathways (Figure 3A, Figure S4A). An interesting and novel finding was that the metabolite sorbitol, a product of aldose reductase and the first step of the polyol pathway, showed to be most responsive to acute exercise in all comparisons. While studies on its effect in skeletal muscle are sparse, some authors have suggested sorbitol can cause hyperosmolar stress, positively affecting glucose uptake [[50], [51]]. Though not reported to be acutely regulated in human muscle before, its proposed function would be consistent with the energy requirements during and following acute exercise. Cluster analysis further revealed differences in how lipid-associated metabolites respond to acute exercise in EG: Following EE, the majority of lipid-associated metabolites were upregulated (8 metabolites; clusters 3 and 4; Figure 3A), compared to 4 metabolites in response to RE (cluster 3) and 3 out of 4 metabolites in the downregulated cluster being lipid-associated (cluster 5). Closer investigation of the lipid superfamily showed derivatives of carnitine oppositely affected in response to EE (4 derivatives up-) and RE (3 derivatives downregulated). The remaining lipid-associated metabolites were regulated in the same direction (Figure 3B,D). In EG, modification of the carnitine shuttle, trafficking long chain fatty acids into the mitochondrion for beta oxidation, constitutes yet another hallmark of energy flux optimization in skeletal muscle tissue. This process results in a reduction in free carnitine and an increase in carnitine derivatives [52]. The ratio between the two has been used as a marker of the utilization of beta-oxidation in metabolic disorders [[53], [54]]. Comparably, a distinct difference in amino acid-associated metabolites comparing EE and RE in SG was observed (Figure 3A, cluster 4, 5, 6; Figure S4A, cluster 4, 5). A closer investigation revealed a large-scale downregulation in response to RE only (Figure 3C), amongst others the branched chain amino acids (BCAA) valine, leucine and isoleucine, but notably not of ketoleucine, a product and indicator of the ongoing BCAA catabolism. In contrast, the only BCAA downregulated in response to EE was valine (Figure 3D). This response pattern and difference between EE and RE is less clear in EG, with all three BCAAs being downregulated in response to EE (Figure S4C). Together, this indicates a sharper, divergent distinction between forms of acute exercise for amino acids (AA) in SG, whereas EG is more optimized for fatty acid trafficking, reflecting exercise background-specific metabolic specializations.

#### 3.4.4 Transcription factor motif activity

To obtain a better understanding of the regulation of the observed transcriptomic changes, we employed Motif Activity Response Analysis (MARA), which pairs transcriptomics with gene-wise TF motif binding site information [[18], [19], [20]]. By doing so, we were able to discover additional exercise-response dynamics. Unlike DEG which peaks at 3h, the most significantly changed TF motifs peaked at the 1h timepoint, primarily increasing in activity (Supplement A, Table S4). Interestingly, some of these comparisons prominently feature HIF1A and MYFfamily. HIF1A is a regulator of mitochondrial metabolism and hypoxia-related processes [[55], [56], [57]], with oxygen delivery to skeletal muscle being one of the primary limiting factors of acute endurance performance [58]. HIF1A controls genes responsible for erythropoiesis and angiogenesis, aimed at coping with and resolving the hypoxic condition [59], but itself relies on a complex regulation involving degradation by the von Hippel-Lindau protein and activation in hypoxic conditions [[60], [61]]. The MYFfamily motif controls the myogenic regulatory factors MYF5, MYF6, MYOD1 and MYOG [62]. Comparing groups following RE, EG was less likely to activate strength adaptation-associated motifs such as MYFfamily but was, amongst others, more likely than SG to activate the HIF1A motif (Supplement A, Table S4). Surprisingly, while RE in EG lead to the highest number of DEGs on transcriptomic level, EE induced a higher number of changes on the TF motif level (Supplement A, Table S4). This difference might be the consequence of a more untargeted, somewhat “chaotic” response to an unfamiliar form of exercise as opposed to a targeted activation of functionally connected genes by specific TFs, and could explain both, transcriptomic and TF level effects. As demonstrated by baseline differences, EG is specialized in a markedly different regulatory direction compared to both CG and SG, including a regulatory specialization with a more pronounced stimulation of EE-associated TFs, such as HIF1A, and thus exhibiting a narrower, but more intense stimulation of transcription as indicated above in the functional cluster analysis (Figure 2C). The same logic could also explain the somewhat blunted activity of the MYFfamily motif following RE in EG compared with CG (Supplement A, Table S4). HIF1A and MYFfamily motifs both show an exercise-specific response in a direct comparison of EE and RE (Figure S6A). Importantly, they seem to only be affected in their specialized activity following EE (HIF1A motif) and RE (MYFfamily motif), but not following the other form of exercise (Figure S6B). Furthermore, we show the CREB1 motif, which regulates PPARGC1A, a master regulator of mitochondrial biogenesis [[63], [64]], to be stimulated by both EE and RE (Figure S4B).

Additionally, we identified the primary genes driving the activity of each motif using correlation analysis (“reverse mapping”), revealing candidate genes highly relevant to their respective motif (Figure S6C), many of which playing a substantial role in the regulatory response to acute exercise: HES1 (associated with HIF1A motif), involved in skeletal muscle differentiation [65], CLIC4 (MYFfamily motif), having a role in angiogenesis [66], TRIM63, a potential regulator of muscle mass [67] and CBFB (both CREB1 motif), a negative regulator of MyoD [68]. Using tissue- and cancer-specific biological network analysis (TCSBN) we further confirmed these candidate genes to be functionally connected rather than a random collection of genes (Figure S6D).

### 3.5 Correlation between physiological, histological and molecular features and effects

**Figure 4:**
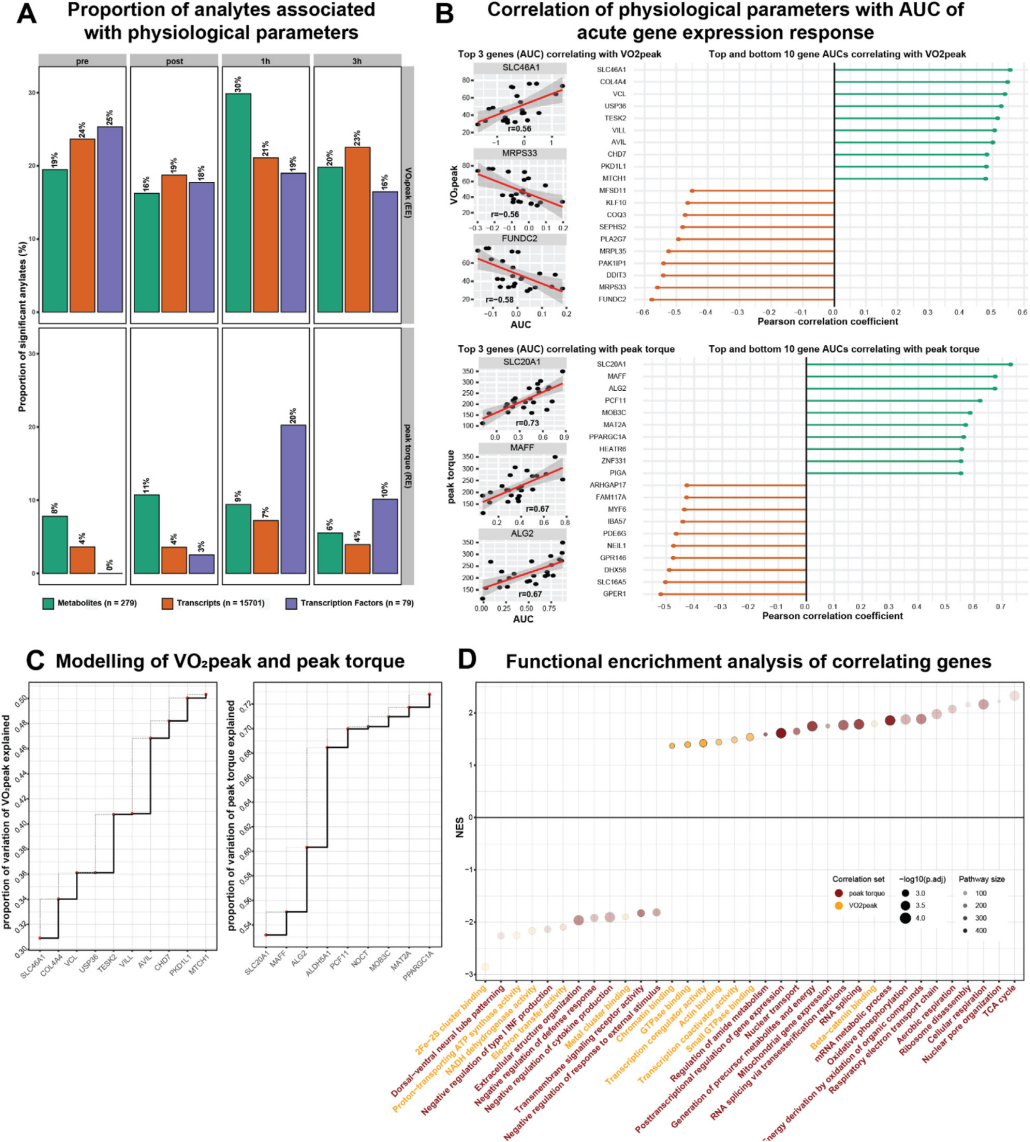
**Correlation of physiological parameters with molecular analytes**. (A) Proportion of analyte response to EE and RE based on area under curve (AUC) significantly correlating to baseline $\dot{V}$O2peak and peak torque respectively. Total number of analytes each in legend parenthesis. (B) Top and bottom 10 genes correlating with $\dot{V}$O2peak and peak torque; top 3 genes shown individually. (C) Modelling of top genes explaining variation of $\dot{V}$O2peak and peak torque. (D) Gene set enrichment analysis of genes (expressed as normalized enrichment score, NES) sorted by correlation for $\dot{V}$O2peak and peak torque.

Molecular processes in skeletal muscle stimulated by acute exercise largely reflect the physical and biochemical requirements of the performance output and the ability of the individual to meet these. To better understand the influence of the individual performance characteristics on skeletal muscle molecular changes at different omics levels following acute exercise, regression analysis was performed between transcripts, metabolites and TFs and $\dot{V}$O2peak (EE) and peak knee torque (RE). Between 16 and 30 % (mean 21 %) of analytes correlated significantly (p < 0.05) with $\dot{V}$O2peak across all timepoints following EE (Figure 4A). In contrast, 0–20 % (mean 7 %) of analytes correlated with peak leg torque following RE. Endurance training has extensive influence on acute mitochondrial activity and ATP production, and subsequently contributes to $\dot{V}$O2peak [69]. RE, on the other hand, stimulates acute molecular mechanisms that will, following a sequence of hypertrophic processes over time, increase peak torque and thus contractile potential. Such a process involves molecular mechanisms such as immune system activation, hypertrophic processes and angiogenesis that take place up to several days following the acute bout [[70], [71]]. To identify genes highly relevant to EE and RE performance via their proxies $\dot{V}$O_2_peak and peak torque, acute gene expression was individually summarized as area under the curve (AUC) over time using trapezoid integration. To further deepen the analysis, significantly correlating genes from the present study were retained only if they were also found to be significantly regulated by acute exercise in both of two large meta-analyses by Amar et al. and Pillon et al. [[43], [72]] (Figure 4B). The present analysis showed a stronger correlation between performance characteristics and genes following RE (up to r = 0.73) than following EE (up to r = −0.58; Figure 4B). Notably, PPARGC1A and myogenesis regulating gene MYF6 in RE were identified in this analysis (Figure 4B). While PPARGC1A has primarily been associated with endurance exercise [[73], [74], [75]], there is some support for a role in resistance exercise, regulating skeletal muscle hypertrophy [[76], [77]]. Muscle regeneration and hypertrophy rely on a choreography of myogenic factors to guide a satellite cell from quiescence to differentiation, with MYF6 activity required only during the terminal differentiation process [78]. It is possible that this negative correlation is an indication of the expression timing of the MYF6 gene. Previous results placed MYF6 activity between 1 and 8 h following acute exercise [79], a range included in the present study. Alternatively, this could be explained by a lower stimulation of pro-hypertrophic processes in the already hypertrophy-adapted SG [80].

Next, we used the identified most highly correlating “top genes” in an attempt to explain inter-individual variance in $\dot{V}$O2peak and peak torque. Surprisingly, results show that as few as 5 transcripts can explain 41 % of the variance of $\dot{V}$O2peak and 4 transcripts 68 % of peak torque variability (Figure 4C). Additionally, to get a better understanding of the processes connected to the identified correlating genes, GSEA was performed for all genes that correlated significantly with peak torque or $\dot{V}$O2peak. Surprisingly, results show a strong positive enrichment of TCA-cycle, cellular respiration and ribosome-related terms in connection to the ability to produce high peak torque such as SG. Enrichment of genes correlating with high $\dot{V}$O2peak was rather focused on receptor binding, transcriptional regulation, the electron transfer chain as well as actin binding (Figure 4D).

### 3.6 Multi-omics molecular clustering

**Figure 5:**
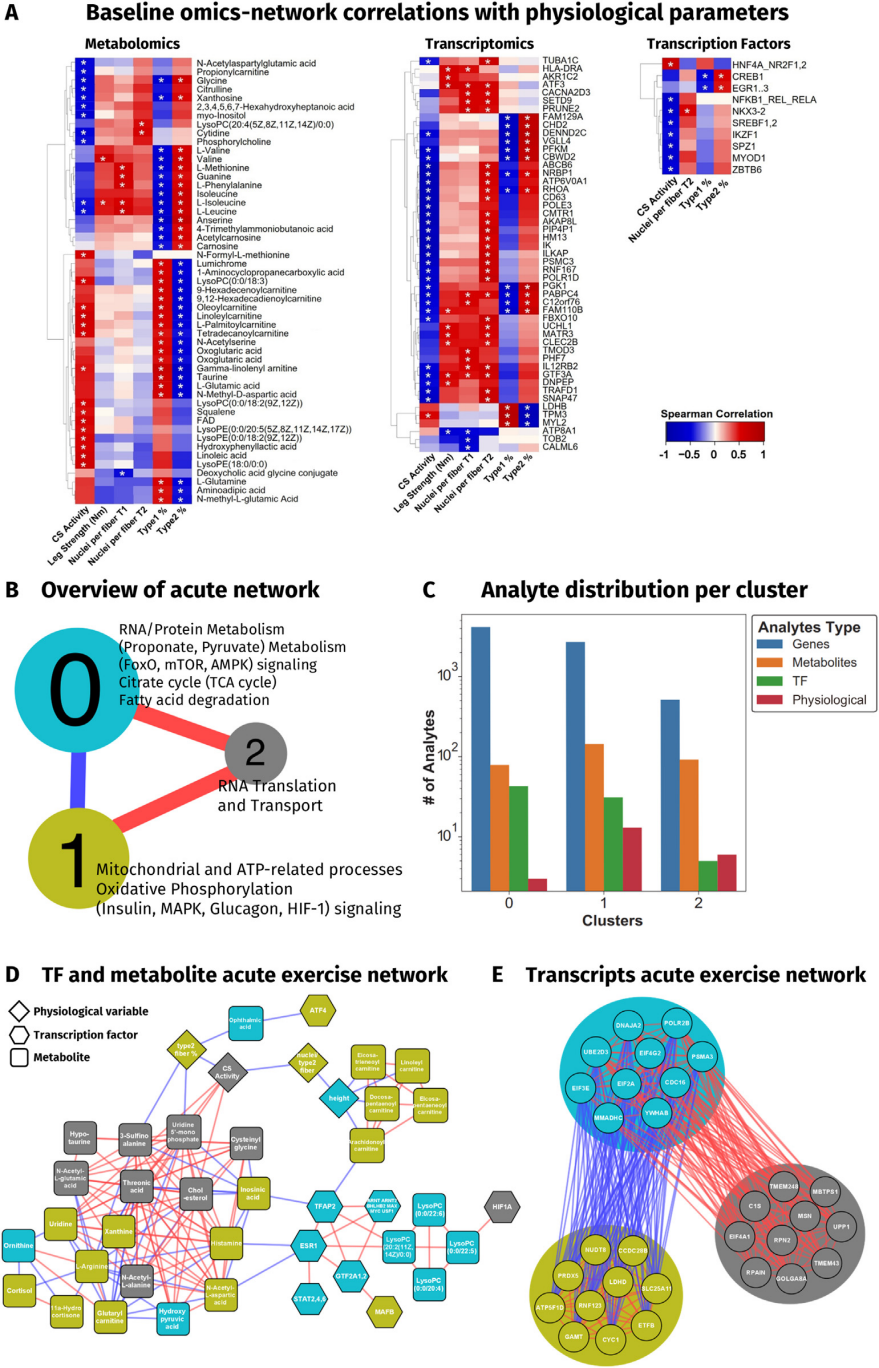
**Omics-network overview and highlights**. (A) Baseline -omics network showing the up to top 10 correlating genes for each physiological variable and all correlating transcription factor motifs and metabolites. (B) Overview over the acute -omics network with functional annotations of the three main clusters. (C) Distribution of analytes within the individual clusters from C. (D) Network of the top 10 % of metabolites, transcription factor motifs and physiological variables from each cluster. Edge colors represent direction and strength of connections. Red = positive, blue = negative. (E) Top 10 genes from each cluster, edges follow the same logic as in D. (fill colors in D and E represent cluster identity as in B; ∗: FDR<0.01 for Spearman correlation).

**Figure 6:**
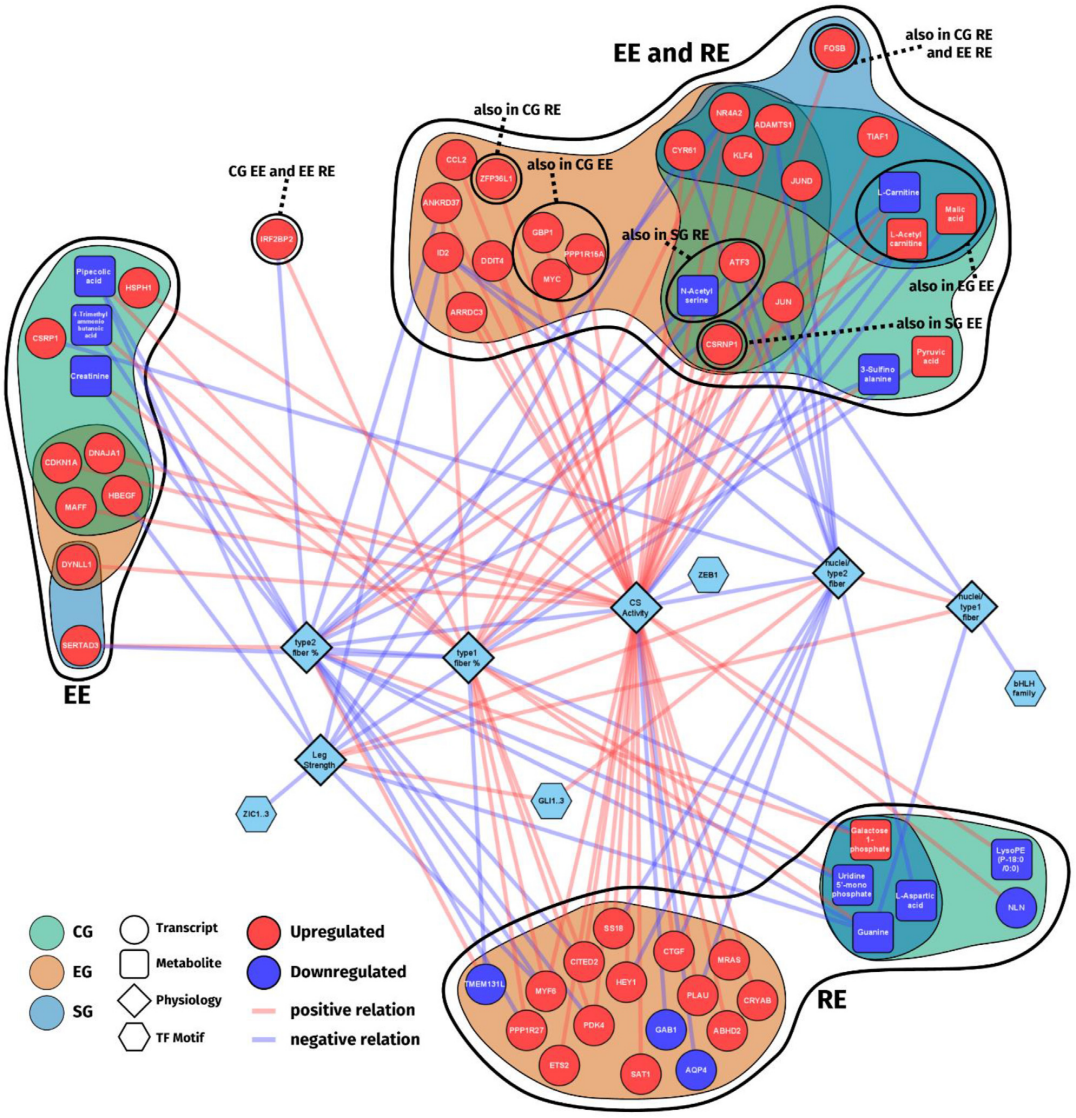
**Multi-omics network of acute exercise**. Multi-omics network of acute exercise showing genes, metabolites and transcription factor motifs significantly changed with acute exercise and positive or negative connection to selected physiological variables grouped by form of acute exercise and subject groups untrained control, and endurance and strength athletes with >15 years of training history.

To obtain a deeper understanding of the relations between -omics data and individual analytes, transcripts, metabolites, clinical and histological data and predicted TF motifs were used for network integration and cluster analysis at baseline (Figure 5) and in response to acute exercise (Figure 5, Figure 6). This network was then used to determine physiological parameter correlations with individual analytes, for cluster identification and functional annotation. The generated network can be interactively accessed and downloaded for cytoscape via https://bit.ly/iNetModels_CrossEX (Multi-omics network → Study-specific Networks → crossEX (Reitzner et al., 2024).

#### 3.6.1 Omics-network at baseline

To analyze the network at baseline we selected physiological, biochemical and histological parameters such as leg strength, CS activity and fiber type composition and analyzed their inter-network connections with the transcriptome, metabolome and TFs. Results show a high number of metabolites correlating with the proportion of type 1 (T1%) and 2 fibers (T2%). In particular, the amino acid group correlated positively with T2% and the fatty acid group of metabolites correlated with T1% (Figure 5A). CS activity was positively correlated primarily with PC- and PE-compounds and four carnitine derivatives, and negatively correlated with amino acids, nucleosides and propionylcarnitine. Such an increased presence of endogenous muscle fatty acids [81], associated with an increase in beta-oxidative capacity has previously been demonstrated [[82], [83]], representing a shift to a more fatty-acid based energy metabolism, enabling EG to utilize the large lipid reserves of the body during long endurance efforts lasting beyond carbohydrate-based reserves. Leg strength showed substantially less correlation to the metabolome at rest, however with the exception BCAAs valine and L-isoleucine. BCAAs are capable of activating mTORC1 and to stimulate muscle protein synthesis [84], a key process for muscle hypertrophy, but are also relevant to muscle regeneration from acute exercise [[85], [86], [87]]. In the transcriptome, results showed a large negative correlation between the expression of genes and parameters associated with EG (CS activity and T1%), while gene expression was largely positively correlated with parameters associated with SG (leg strength, myonuclear number and T2%) (Figure 5A). It is not unlikely that this, to a large degree, can be explained by the increased at-rest potential capacity of RNA synthesis in SG through the observed higher proportion of myonuclei per muscle fiber (Table 1). One notable exception to the general trend of negative correlation in EG was TPM3, which was positively correlated with a greater T1% and previously reported as involved in the calcium-dependent regulation of slow-twitch skeletal muscle fiber, significantly contributing to their contractile force [88]. For TF motifs we observed the same negative trend of most notably the CREB1 motif correlation with T1%, but an increased motif activity with T2% (Figure 5A). CREB1 mediates PPARGC1A activity, indicating a potential blunted activity in subjects with high T1% - endurance athletes with already highly adapted mitochondrial machineries.

#### 3.6.2 Omics-network of the acute exercise response

To better define the structure of the acute network we created, we performed community detection analysis using Leiden clustering algorithm [89]. We identified three main clusters after maximizing the modularity score of all types of analytes (Figure 5B), providing a more comprehensive understanding of the general processes taking place in our created network. Results showed the biggest cluster (cluster 0) to be associated with RNA and protein metabolism, FoxO, mTOR and AMPK signaling, the TCA cycle and fatty acid degradation. In animal models, FoxO signaling has previously been shown to improve glucose and lipid metabolic control via gluconeogenic enzymes [[90], [91], [92], [93]] and lipase activity [90], consequently increasing metabolic control. Furthermore, it has been implicated in the process of muscle mass maintenance and in controlling the myogenic factors in humans, extensively reviewed by Gross et al. [94]. Similarly, mTOR is a key mediator of muscle protein synthesis [[95], [96]] and is stimulated by resistance exercise to a higher extent than by endurance exercise [97]. AMPK is a metabolic master switch, downregulating anabolic-, and upregulating catabolic processes, as well as inducing mitochondrial biogenesis [[98], [99]]. Also, it is influenced by being inhibited via acute exercise-reduced phosphocreatine [100], but also long-term via TFs such as, GEF and MEF2 and the transcriptional coactivator PPARGC1A [101]. In combination with the other functions of cluster 0, these key regulatory axes can dictate metabolic status and trajectory in skeletal muscle. Cluster 0 was negatively correlated to cluster 1, which was associated with mitochondrial and ATP-related processes, oxidative phosphorylation and insulin-, MAPK-, glucagon- and HIF-1 signaling. Again, energy metabolism is central to this cluster, with anabolic, catabolic and amphibolic elements, such as MAPK which acts via targets such as PPARGC1A, ATF2 and CREB [102]. In addition to the above-mentioned systemic role of HIF-1A, locally it can indicate increased hypoxia as a consequence of increased oxygen-consuming ATP provision [103]. Both these clusters were positively correlated to cluster 2, associated with RNA transport and translation into proteins. Due to the overrepresentation of transcriptomic analytes (Figure 5C), analytes with the highest degree of connection are shown separated into top 10 % TFs and metabolites (Figure 5D), and top 10 transcripts (Figure 5E). Here, metabolites of cluster 2 showed a higher degree of centrality, supporting cluster 2's central role in driving protein translation for pathways of both the other clusters. Furthermore, on individual analyte level, reiterating the most central pathways of cluster 1 in Figure 5B, six of the top 10 genes (SLC25A11, NUDT8, LDHD, ATP5F1D, CYC1, ETFB) of cluster 1 are mitochondrial protein-coding genes that are part of the electron transport chain or the TCA cycle.

To contextualize the acute exercise network with measures of performance, we selected EG- and SG-representative parameters such as CS activity, leg strength and T1% and T2%. Then we selected only analytes (transcripts, metabolites, TFs) that were significantly correlated with these parameters, filtered the resulting network with only analytes that were significantly changed with acute exercise and grouped them by acute exercise and subject group. In Figure 6, genes that are both significantly affected by training background and acute exercise, reinforcing their high relevance for exercise training, are visualized. Most analytes were connected to CS activity or T2%, the two physiological parameters with the highest degree of centrality. EG showed to be the most affected group, specifically in the type of exercise they were not used to (RE). In a functional analysis using gene ontology in EG, RE exclusively stimulated genes significantly associated with anatomical structure formation involved in morphogenesis and the response to hormones. Most notable were previously mentioned MYF6, CITED2, an inhibitor of HIF1A signaling and modulator of PPARGC1A [[104], [105], [106]], and PDK4, a mitochondrial protein that redirects the metabolic flux from glycolysis to fat metabolism [107]. This high number of RE responsive genes in EG compared to SG suggest a higher potential transcriptomic stimulation potential in response to the form of exercise they are not used to. Furthermore, the RE-stimulated transcripts in EG were primarily associated with CS activity rather than RE-related performance parameters such as T2%. This suggests a fundamentally different molecular response when performing RE in endurance trained compared to strength trained individuals. The 5 identified core genes responsive to both EE and RE in all three groups were transcription factor and growth factor-related genes, one of which, AMATS1 has been shown to interact with the growth factor VEGFA [108], suggesting a common, exercise-independent growth and TF signaling program.

## 4 Discussion

In a highly controlled human exercise intervention study, multi-omics network integration analysis of the response to acute endurance and resistance exercise revealed distinct differences between the two exercise types. We also found clear differences in the acute exercise molecular responses between long-term high-level athletes, particularly endurance athletes, and control subjects with an exercise-specific specialization of molecular mechanisms. Specifically, we show that endurance athletes increase their proficiency in the fatty acid metabolism, increasing the abundance of carnitine-derivate metabolites, and uniquely down-regulate genes related to mitochondria, transcription and ribosomes, while strength athletes change the dynamics of their amino acid metabolism, increase the abundance of unsaturated phospholipid metabolites but on transcriptional level stay closer to control subjects than endurance athletes do. Additionally, results suggest a “transcriptional specialization effect” by transcriptional narrowing and intensification depending on the type of exercise training performed. This is complemented by altered transcription factor activity, particularly of HIF1A and MYF-family members.

### 4.1 Training-background specific adaptations of energy metabolism

On metabolic level, carnitines, chaperone molecules of beta-oxidation, and fatty acids of different lengths bound to these carnitines were enriched in EG, while in SG PCs and PEs were enriched, contributing to membrane fluidity and dynamics of, amongst others, GLUT4, improving glucose uptake potential. A key adaptation to exercise training lies in the way ATP is produced, which in the endurance group (EG) was reflected in the transcriptomic enrichment of mitochondrial and energy metabolic pathways at baseline compared to both the other groups. While it has to be kept in mind that to a certain level genetic predetermination effects can play a role in such adaptational potential, those findings are likely strongly dependent on a higher CS activity and mitochondrial volume. Such metabolic adaptations reflect an improvement of the major energy metabolic systems – with beta-oxidation improvements in EG and glucose metabolism improvements in SG. Unexpectedly, the acute exercise response revealed a steep downregulation of both energy metabolic pathways and transcriptomics-based metabolic flux in EG only. A sharp immediate decrease in energy metabolic pathways upon cessation of acute exercise suggests a tight regulatory control and a focus on providing energy to exercise tasks only. In agreement with this, previous studies have shown EG adaptation to largely lie in energy metabolic optimization, providing a high flux of ATP over sessions of extended exercise [109]. This notion is additionally supported by EG's and SG's metabolic response to EE and RE, respectively. Specifically, emphasizing carnitine metabolism in endurance athletes performing EE and AA metabolism in resistance-trained athletes performing RE suggests a highly specific metabolic specialization to the familiar form of exercise: Since we show that carnitine-derivatives in EG are largely upregulated following EE, and largely downregulated following RE, a distinction not found in CG and SG, and that AAs are primarily downregulated in SG performing RE, but not following EE, this subsequently allows for physical performance beyond the levels of untrained individuals. One example of the ad-hoc upregulation of energy flux common across all groups is the metabolite sorbitol, aiding glucose uptake into the cell, which we, for the first time in humans, report to be upregulated in response to EE and RE.

Our integrated network analysis revealed further evidence highlighting the core role of an exercise-adjusted energy metabolism. Results show it to be central player in both forms of acute exercise with the citrate cycle, FA and protein metabolism, and insulin and glucagon signaling prominently represented in cluster annotations (Figure 5B). That carnitine metabolites correlate with high aerobic capacity, a proxy for endurance performance, and amino acid metabolites correlate with strength parameters reflect substantial energy metabolism-related group differences, observed in the baseline network. Particularly, one of the genes exclusively regulated in EG in response to RE was PDK4, a gene responsible for orchestrating metabolic flux, further reinforcing the notion of the EG specialization towards optimization of ATP production. Together, our findings demonstrate background-specific metabolic adaptation, resulting in increased energy metabolic control as a consequence of prolonged, in our study >15 years, of exercise-form specific training, adding to our understanding of high-level athlete adaptations and underlining the substantial uniqueness of EG.

### 4.2 Adaptational potential is retained in highly specialized athletes

Several findings in this current study support the notion of a retained high potential for resistance exercise adaptation in EG compared to SG: In the created acute network we showed high numbers of genes exclusively regulated in EG in response to RE which are highly relevant for RE-specific adaptation processes. Apart from the above mentioned PDK4 it also included MYF6 and CITED2, regulating hypertrophy and angiogenesis (Figure 6). Additionally, all groups significantly increased HIF1A and MYFfamily TF activity in response to EE and RE, respectively. This is noteworthy because both of their gene targets are early activated in the regulatory cascade of exercise-specific adaptation [[61], [62]]. Beyond this, following an acute bout of exercise they are not familiar with, both athlete groups retain a higher number of DEGs. Additionally, regulation of EE and RE follows an exercise type-specific pattern, with an increase in HIF1A and MYFfamily TF motif activity respectively, despite the above-mentioned group-specific specializations. This indicates that form of acute exercise rather than the training history is the largest determinant of the individual exercise session response. These notions are supported by indications from previous publications: Exercise-naïve individuals have been shown to exhibit a prolonged elevation of muscle protein synthesis following a bout of acute resistance exercise [110]. It has also been shown that previous resistance exercise experience increasingly protects against muscle damage from subsequent bouts [80], laying the foundation for the idea of a retainment of adaptational potential even in highly specialized, though RE-naïve EG.

### 4.3 Long-term training induces molecular specialization by transcriptional amplification and narrowing

Gene clusters summary response was substantially *stronger* expression (higher fold-change) in the familiar form of exercise, however following the same general response shape (Figure 2C). However, at the 3h timepoint, we found *fewer* DEGs in athletes performing their familiar form of exercise compared to their exercise-naïve peers (Figure 1C). While it might be tempting to accredit this to a simple blunting of gene expression effect, together with the abovementioned increase in the response amplitude, alternatively this can be accredited to an *optimized* response in the respective experienced athletes. Following acute exercise, DEGs increased from post to the 3h timepoint and were increasingly dominated by regeneration-related pathways, representing a commonly observed optimization of regeneration from acute exercise with long-term training [111]. However, we showed the gene clusters in response to EE and RE to be strongly associated with more adaptational pathways such as transcriptional regulation and translation, and muscle filament-related processes. Additionally, only RE, not EE, was associated with myoblast differentiation, growth, and morphogenesis. Taken together, these observations suggest the difference between subject groups is based on the degree of gene activation rather than number of DEGs. In agreement with the presented results, it has previously been indicated that muscle memory effects are mediated by modification of the translational capacity, and thus potential amplitude of protein expression in the context of acute exercise [112]. Additional observations support the idea of an exercise-specific trajectory of development from untrained to trained state via degree of molecular and metabolic exercise-specific optimization: On metabolomic level in EG, lipid-associated metabolites were reduced in response to RE but not EE (Figure 3A). In SG however, RE largely decreased amino acid-instead of lipid-associated metabolites, indicating either a lower usage of lipids as a source of energy or altered lipid synthesis dynamics in SG.

#### 4.3.1 Performance diversity can be explained by small numbers of genes

By correlating differential gene expression and physiological performance parameters, we showed, that only 4 genes can explain 68 % of the leg strength variation and 5 genes 42 % of $\dot{V}$O2peak. Interestingly, GSEA analysis associated the RE-correlating genes with energy metabolism and transcription and translation processes. Despite being less studied, RE requires and results in substantial energy metabolic perturbations [113]. Together, this might underline the importance of further research into the resistance exercise-related energy metabolism. On the other hand, the broad influence of $\dot{V}$O2peak results in a higher *number* of significantly correlating genes, mainly associated with transcription processes and their regulation. Leg strength correlates stronger with individual genes as it is a closer representation of *local* performance and thus more adequately representing *local* gene expression, in particular energy metabolic pathways important for and perturbed by acute exercise.

### 4.4 In-silico methods add to the understanding of complex data

Metabolic flux related to the citrate cycle and beta-oxidation showed to be distinctively down-regulated in EG only following EE as analyzed by genome-scale metabolic modelling (GEM), indicating EGs particularly tight metabolic control over energy metabolic pathways as discussed above. Additionally, we identified HES1, CLIC4 and TRIM63 as key targets of HIF1A and MYFfamily TF motifs following EE and RE respectively, demonstrating a robust form of exercise-specific gene regulatory program independent of exercise background. These findings demonstrate the ability of both these in-silico methods to enhance the perspective on transcriptomic data by adding database-originating layers of information.

### 4.5 Limitations

While the criteria used to include subjects to this study were based on both training history and performance phenotype, non-physiological parameters such as social, cultural as well as genetic factors could influence the observed molecular variations between groups. What we show here is how individuals with high $\dot{V}$O_2_peak or leg strength and a long history of training differ from each other and from untrained controls on molecular level. The fact that all subjects in SG were stronger than any of the subjects in the other groups and all subjects in EG had higher $\dot{V}$O_2_peak do not necessarily mean that this was a consequence of the conducted exercise training alone. Nevertheless, high-volume and -intensity exercise training over 15 years leads to significant adaptation in addition to any baseline untrained performance level. While it can safely be assumed that a large fraction of the molecular differences between groups are dependent on the very different training regimens prior to study inclusion, the abovementioned parameters should be kept in mind when interpreting molecular data in human exercise studies. Irrespective of such influence, the large effect of structured progressive training as performed by the athletes in the present study provides a strong foundation for the rationale to investigate baseline and acute post-exercise differential omics analyses in human subjects.

## 5 Conclusions

Highly endurance trained athletes specialize in lipid-related energy processes while resistance trained athletes change the way they utilize AAs, a diverging development from the untrained state visible from omics and network perspectives. In the process of doing so, for the first time we identified the metabolite sorbitol as substantially upregulated with acute exercise. Additionally, endurance athletes increase the abundance of carnitine-derivate metabolites and strength athletes the abundance of unsaturated phospholipid metabolites. We further enhanced our analysis using GEM and MARA analysis, showing a striking down-regulation of metabolic flux as a consequence of EGs training-induced metabolic adaptation also reflected in the transcriptomic results, and identifying the HIF1A and MYFfamily TF motif as exclusive to EE and RE respectively, independent of exercise background. Together, these findings put forward the concept of a numerically smaller but more intense stimulation of genes – a transcriptional specialization - with long-term high-level training, a metabolic specialization focused on the needs of the type of exercise training performed, and a certain degree of transcription factor motif specificity to the form of acute exercise, driven by particular sets of exercise-responsive genes.

Multi-omics and large data approaches are increasingly used for precision health and physical activity research. Here, we employed such an approach to compute differences between high-level endurance and strength athletes with at least 15 years of training history and untrained individuals, showing major differences in energy metabolic and amino acid-related pathways following acute exercise, and providing a publicly available resource of transcriptomic, metabolomic and regulomic data to be used for further investigations into the molecular biology of exercise.

## CRediT authorship contribution statement

**Stefan M. Reitzner:** Conceptualization, Data curation, Formal analysis, Funding acquisition, Investigation, Methodology, Project administration, Resources, Software, Validation, Visualization, Writing – original draft, Writing – review & editing. **Eric B. Emanuelsson:** Data curation, Investigation, Methodology, Resources, Writing – review & editing. **Muhammad Arif:** Data curation, Formal analysis, Software, Writing – review & editing. **Bogumil Kaczkowski:** Investigation, Software, Supervision, Writing – review & editing. **Andrew TJ. Kwon:** Investigation, Software, Supervision, Conceptualization, Writing – review & editing. **Adil Mardinoglu:** Software, Supervision, Writing – review & editing. **Erik Arner:** Investigation, Software, Supervision, Writing – review & editing. **Mark A. Chapman:** Investigation, Supervision, Writing – review & editing. **Carl Johan Sundberg:** Conceptualization, Funding acquisition, Investigation, Project administration, Supervision, Writing – review & editing.

## Declaration of competing interest

The authors declare that they have no known competing financial interests or personal relationships that could have appeared to influence the work reported in this paper.

## Data Availability

Data will be made available on request.
