# Supplementary material for: Molecular profiling of high-level athlete skeletal muscle after acute endurance or resistance exercise – A systems biology approach
Source: Mol Metab. 2023 Dec 21;79:101857. doi: 10.1016/j.molmet.2023.101857 (PMC10805945; doi:10.1016/j.molmet.2023.101857)
Supplement: Multimedia component 1 [file mmc1.docx]

| *vs pre →* | Pre (EE) | Pre (RE) | Post (EE) | Post (RE) | 1h (EE) | 1h (RE) | 3h (EE) | 3h (RE) |
| --- | --- | --- | --- | --- | --- | --- | --- | --- |
| groups pooled (n=24) vs pre |  |  | 283 | 226 | 1771 | 2063 | 2691 | 4542 |
| *EE vs RE* | *0* | | *44* | | *509* | | *1783* | |
| control (n=8) vs pre |  |  | 97 | 60 | 571 | 571 | 1950 | 2227 |
| *EE vs RE* | *0* | | *7* | | *96* | | *412* | |
| endurance (n=8) vs pre |  |  | 106 | 76 | 486 | 609 | 717 | 2515 |
| *EE vs RE* | *0* | | *8* | | *73* | | *500* | |
| strength (n=8) vs pre |  |  | 66 | 46 | 723 | 1007 | 1121 | 2314 |
| *EE vs RE* | *0* | | *3* | | *99* | | *344* | |
|  |  |  |  |  |  |  |  |  |
| *between groups comparison* | Pre (EE) | Pre (RE) | Post (EE) | Post (RE) | 1h (EE) | 1h (RE) | 3h (EE) | 3h (RE) |
| control vs endurance | 218 | 228 | 21 | 333 | 88 | 1043 | 122 | 175 |
| *pooled EE+RE* | *1363* | | *1159* | | *1697* | | *897* | |
| control vs strength | 3 | 2 | 21 | 8 | 4 | 101 | 1 | 6 |
| *pooled EE+RE* | *57* | | *185* | | *506* | | *27* | |
| strength vs endurance | 40 | 192 | 6 | 159 | 14 | 313 | 18 | 150 |
| *pooled EE+RE* | *919* | | *569* | | *608* | | *453* | |
|  |  | |  | |  | |  | |
| *Intersections of ↓ / vs pre→* | Pre (EE) | Pre (RE) | Post (EE) | Post (RE) | 1h (EE) | 1h (RE) | 3h (EE) | 3h (RE) |
| control ∩ endurance |  |  | 59 | 39 | 283 | 316 | 528 | 1505 |
| control ∩ strength |  |  | 44 | 38 | 319 | 397 | 762 | 1518 |
| strength ∩ endurance |  |  | 44 | 31 | 257 | 379 | 378 | 1507 |
| control ∩ endurance ∩ strength |  |  | 36 | 30 | 202 | 275 | 350 | 1214 |
| pooled ∩ pooled |  | | 129 | | 1043 | | 1836 | |
| control ∩ control |  | | 44 | | 254 | | 869 | |
| endurance ∩ endurance |  | | 41 | | 194 | | 433 | |
| strength ∩ strength |  | | 32 | | 389 | | 649 | |

**A**

**C**

**B**

*196*

*91*

*21*

Table S1: Number of **differentially expressed genes** (DEG) in the three dimensions (time, group, acute intervention). **A**: Within-group comparisons. Numbers in the first row of each section refer to the comparison pre versus the respective timepoint, the second row of each block refers to the comparison between acute endurance (EE) and acute resistance (RE) exercise. In the first block, numbers represent DEG of all subject groups combined. **B**: Between-group comparisons. In the first row of each section separated by acute intervention, in the second row of each section both acute interventions pooled together. **C**: The genes that sets of DEG have in common based on the pre vs later timepoint comparison. In the first section comparing subject groups within their acute intervention, in the second section comparing acute interventions within each subject group. Braces indicate the intersect of the values they summarize. (FDR < 0.01 and FC>1.25 or <0.8)

| *vs pre →* | Pre (EE) | Pre (RE) | Post (EE) | Post (RE) | 1h (EE) | 1h (RE) | 3h (EE) | 3h (RE) |
| --- | --- | --- | --- | --- | --- | --- | --- | --- |
| groups pooled (n=24) vs pre |  |  | 59 | 75 | 31 | 44 | 18 | 28 |
| *EE vs RE* | *1* | | *51* | | *38* | | *17* | |
| control (n=8) vs pre |  |  | 41 | 44 | 16 | 15 | 6 | 13 |
| *EE vs RE* | *2* | | *18* | | *12* | | *12* | |
| endurance (n=8) vs pre |  |  | 37 | 23 | 12 | 14 | 7 | 11 |
| *EE vs RE* | *0* | | *27* | | *14* | | *16* | |
| strength (n=8) vs pre |  |  | 20 | 60 | 18 | 35 | 8 | 20 |
| *EE vs RE* | *6* | | *37* | | *23* | | *7* | |
|  |  |  |  |  |  |  |  |  |
| *between groups comparison* | Pre (EE) | Pre (RE) | Post (EE) | Post (RE) | 1h (EE) | 1h (RE) | 3h (EE) | 3h (RE) |
| control vs endurance | 44 | 43 | 39 | 63 | 55 | 26 | 34 | 48 |
| *pooled EE+RE* | *77* | | *94* | | *82* | | *68* | |
| control vs strength | 14 | 14 | 13 | 10 | 10 | 2 | 1 | 6 |
| *pooled EE+RE* | *34* | | *33* | | *26* | | *20* | |
| strength vs endurance | 85 | 53 | 46 | 24 | 24 | 45 | 36 | 66 |
| *pooled EE+RE* | *115* | | *105* | | *116* | | *100* | |
|  |  | |  | |  | |  | |
| *Intersections of ↓ / vs pre→* | Pre (EE) | Pre (RE) | Post (EE) | Post (RE) | 1h (EE) | 1h (RE) | 3h (EE) | 3h (RE) |
| control ∩ endurance |  |  | 28 | 19 | 5 | 7 | 3 | 5 |
| control ∩ strength |  |  | 18 | 36 | 10 | 14 | 3 | 12 |
| strength ∩ endurance |  |  | 18 | 20 | 5 | 13 | 2 | 8 |
| control ∩ endurance ∩ strength |  |  | 18 | 19 | 5 | 7 | 2 | 5 |
| pooled ∩ pooled |  | | 39 | | 21 | | 13 | |
| control ∩ control |  | | 28 | | 6 | | 2 | |
| endurance ∩ endurance |  | | 19 | | 5 | | 2 | |
| strength ∩ strength |  | | 18 | | 11 | | 8 | |

**A**

**C**

**B**

*0*

*2*

*9*

Table S2: Number of regulated **metabolites** in the three dimensions (time, group, acute intervention). **A**: Within-group comparisons. Numbers in the first row of each section refer to the comparison pre versus the respective timepoint, the second row of each block refers to the comparison between acute endurance (EE) and acute resistance (RE) exercise. In the first block, numbers represent metabolites of all subject groups combined. **B**: Between-group comparisons. In the first row of each section separated by acute intervention, in the second row of each section both acute interventions pooled together. **C**: The metabolites that sets of metabolites have in common based on the pre vs later timepoint comparison. In the first section comparing subject groups within their acute intervention, in the second section comparing acute interventions within each subject group. Braces indicate the intersect of the values they summarize. (FDR < 0.05)

|  |  |  |  |  |  |  |  |  |
| --- | --- | --- | --- | --- | --- | --- | --- | --- |
| *vs pre →* | Pre (EE) | Pre (RE) | Post (EE) | Post (RE) | 1h (EE) | 1h (RE) | 3h (EE) | 3h (RE) |
|  |  |  |  |  |  |  |  |  |
| groups pooled |  |  | ↑88 ↓83 | ↑88 ↓53 | ↑182 ↓1 | ↑55 ↓34 | ↑371 ↓8 | ↑357 ↓49 |
| *EE vs RE* | *↑2 ↓0* | | *↑4 ↓3* | | *↑25 ↓10* | | *↑83 ↓22* | |
| control |  |  | ↑44 ↓1 | ↑31 ↓66 | ↑41 ↓0 | ↑13 ↓9 | ↑417 ↓0 | ↑218 ↓22 |
| *EE vs RE* | *↑0 ↓22* | | *↑1 ↓0* | | *↑9 ↓2* | | *↑19 ↓21* | |
| endurance |  |  | ↑30 ↓332 | ↑4 ↓12 | ↑178 ↓4 | ↑26 ↓17 | ↑317 ↓9 | ↑226 ↓2 |
| *EE vs RE* | *↑5 ↓0* | | *↑2 ↓3* | | *↑28 ↓3* | | *↑2 ↓14* | |
| strength |  |  | ↑54 ↓8 | ↑32 ↓0 | ↑17 ↓3 | ↑21 ↓20 | ↑361 ↓0 | ↑420 ↓0 |
| *EE vs RE* | *↑39 ↓0* | | *↑118 ↓0* | | *↑35 ↓1* | | *↑55 ↓28* | |
|  |  |  |  |  |  |  |  |  |
|  | Pre (EE) | Pre (RE) | Post (EE) | Post (RE) | 1h (EE) | 1h (RE) | 3h (EE) | 3h (RE) |
| *group comparison* |  |  |  |  |  |  |  |  |
|  |  |  |  |  |  |  |  |  |
| control vs endurance | ↑0 ↓1016 | ↑14 ↓805 | ↑3 ↓1027 | ↑2 ↓966 | ↑3 ↓1213 | ↑4 ↓852 | ↑2 ↓906 | ↑14 ↓829 |
| *pooled EE+RE* | *↑6 ↓1019* | | *↑3 ↓1129* | | *↑9 ↓1116* | | *↑14 ↓967* | |
| control vs strength | ↑0 ↓69 | ↑269 ↓2 | ↑2 ↓32 | ↑149 ↓17 | ↑2 ↓21 | ↑3 ↓23 | ↑8 ↓2 | ↑0 ↓41 |
| *pooled EE+RE* | *↑0↓20* | | *↑83 ↓43* | | *↑2 ↓27* | | *↑2 ↓156* | |
| strength vs endurance | ↑0 ↓933 | ↑14 ↓890 | ↑0 ↓957 | ↑0 ↓1114 | ↑8 ↓1096 | ↑2 ↓1171 | ↑0 ↓840 | ↑7 ↓663 |
| *pooled EE+RE* | *↑9↓1054* | | *↑0 ↓1216* | | *↑10 ↓1313* | | *↑11 ↓850* | |

Table S3: Number of regulated **reporter metabolites** identified through GEM analysis. ↑are number of metabolites expressed higher in the specified comparison, ↓ are number of metabolites expressed lower in the comparison. **A**: Metabolites regulated in a time course comparison (post exercise timepoints vs pre; first row of each block) and comparing acute form of exercise (acute endurance exercise = EE vs acute resistance exercise = RE; second row of each block). **B**: Metabolites expression difference comparing subject groups separated by form or acute exercise (first row of each block) and acute forms of exercise pooled (second row of each block).

**A**

**B**

| *timepoint comparison (A vs B)* | *motif up in A (#)* | *motif up in B (#)* |
| --- | --- | --- |
| control / EE / pre vs post | ELK1,4 GABP; (1) | TBP, ATF5 CREB3; NFKB1 REL RELA; ATF4; JUN; IKZF1; SOX17; EBF1; (8) |
| control / RE / pre vs post | ∅ | ATF5 CREB3; JUN; (2) |
| endurance / EE / pre vs post | ∅ | TBP; ATF5 CREB3; NFKB1 REL RELA; JUN; SOX17; (5) |
| endurance / RE / pre vs post | ∅ | TBP; ATF5 CREB3; JUN; NFKB1 REL RELA; (4) |
| strength / EE / pre vs post | ∅ | JUN; (1) |
| strength / RE / pre vs post | ∅ | JUN; (1) |
| control / EE / pre vs 1h | ∅ | ATF5 CREB3; TBP; ATF4; STAT2,4,6; JUN; SOX17; IKZF1; NFKB1 REL RELA; CREB1; GTF2I; NFY; (11) |
| control / RE / pre vs 1h | NRF1; (1) | TBP; ATF5 CREB3; JUN; ATF4; SOX17; IKZF1; STAT2,4,6; NFKB1 REL RELA; MYFfamily; (9) |
| endurance / EE / pre vs 1h | ∅ | ATF5 CREB3; ATF4; JUN; STAT2,4,6; SOX17; CREB1; (6) |
| endurance / RE / pre vs 1h | ∅ | ATF5 CREB3; ATF4; JUN; NKX3-2; SOX17; NFKB1 REL RELA; TFAP4; SPI1; (8) |
| strength / EE / pre vs 1h | ∅ | ATF5 CREB3; STAT2,4,6; JUN; (3) |
| strength / RE / pre vs 1h | ELF1,2,4; NRF1; ELK1,4 GABP{A,B1}; CTCF; (4) | ATF5 CREB3; ATF4; JUN; STAT2,4,6; NFKB1 REL RELA; SOX17; IKZF1; GTF2I; MYFfamily; EGR1-3; (10) |
| control / EE / pre vs 3h | RXR{A,B,G}; (1) | ATF5 CREB3; SREBF1,2; ATF4; RBPJ; (4) |
| control / RE / pre vs 3h | NFR1; RFX1-5 RFXANK RFXAP; GFI1; EHF; RXR{A,B,G}; ELK1,4 GABP{A,B1}; (6) | ATF5 CREB3; JUN, ATF4; TFCP2; (4) |
| endurance / EE / pre vs 3h | ∅ | JUN; CREB1; (2) |
| endurance / RE / pre vs 3h | NRF1; GFI1; ELK1,4 GABP{A,B1}; RXR{A,B,G}; (4) | ATF5 CREB3; JUN; ATF4; NKX3-2; MYOD1; CREB1; MYFfamily; (7) |
| strength / EE / pre vs 3h | ∅ | SREBF1,2; (1) |
| strength / RE / pre vs 3h | NRF1; EHF; RFX1-5 RFXANK RFXAP; GFI1; ELK1,4 GABP{A;B1}; RXR{A,B,G}; IRF1,2,7; (7) | ATF5 CREB3; ATF4; JUN; CREB1; IKZF1; (5) |
| *group comparisons (A vs B)* | *motif up in A (#)* | *motif up in B (#)* |
| 1h / RE / endurance vs control | HNF4A NRF2F1,2; (1) | TBP; TLX1-3 NFIC; TP53; ZIC1-3; JUN; STAT2,4,6; ZNF423; IKZF1; NFKB1 REL RELA; LMO2; SMAD1‑7,9; SPZ1; SPIB; MTF1; MYFfamily; TFAP4; NFIX; EP300; PATZ1; KLF4; UFEwm; (21) |
| 1h / RE / strength vs control | ∅ | SNAI1-3; (1) |
| 1h / RE / strength vs endurance | ∅ | PAX2; (1) |
| 3h / EE / endurance vs control | ∅ | IKZF1, MYFfamily; (2) |
| 3h / RE / strength vs endurance | ∅ | NFKB1 REL RELA; IKZF1; HIF1A; (3) |
| intervention comparisons (A vs B) | *motif up in A (#)* | *motif up in B (#)* |
| 1h / endurance / EE vs RE | STAT2,4,6; (1) | ∅ |
| 3h / control / EE vs RE | ELF1,2,4; ELK1,4 GABP{A,B1}; EHF; RBPJ; SREBF1,2; (5) | ∅ |
| 3h / endurance / EE vs RE | SREBF1,2; (1) | MYFfamily; (1) |

Table S4: **Transcription factor motif activity** significantly different in timepoint, group and acute intervention comparisons. First column shows the comparison made. Second column shows transcription factor motifs upregulated in group A and number of motifs. Third column shows transcription factor motifs upregulated in group B. Number of motifs in parentheses. (FDR < 0.05)
