# Supplementary material for: Molecular profiling of high-level athlete skeletal muscle after acute endurance or resistance exercise – A systems biology approach
Source: Mol Metab. 2023 Dec 21;79:101857. doi: 10.1016/j.molmet.2023.101857 (PMC10805945; doi:10.1016/j.molmet.2023.101857)
Supplement: Multimedia component 5 [file mmc5.docx]

**SUPPLEMENTARY FIGURES**


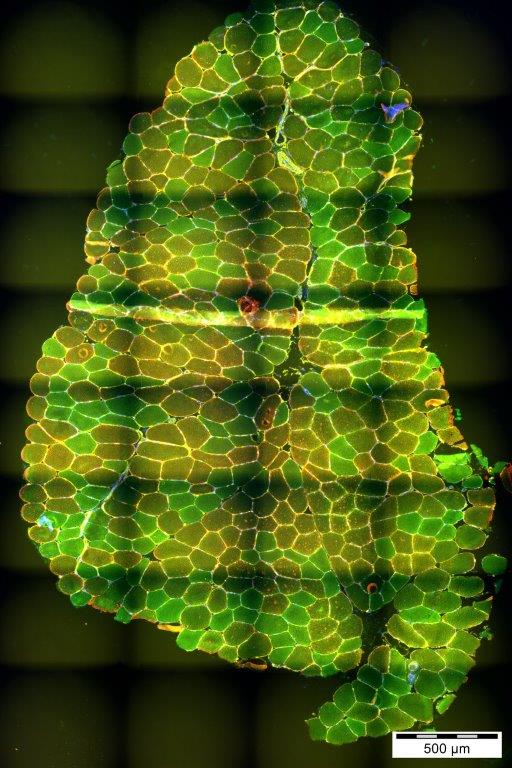


**Figure S1: Histological analysis of skeletal muscle**

*Representative immunohistochemical staining of M. vastus lateralis. Staining was performed for Laminin (yellow), type I fibers (green) and type II fibers (red).*


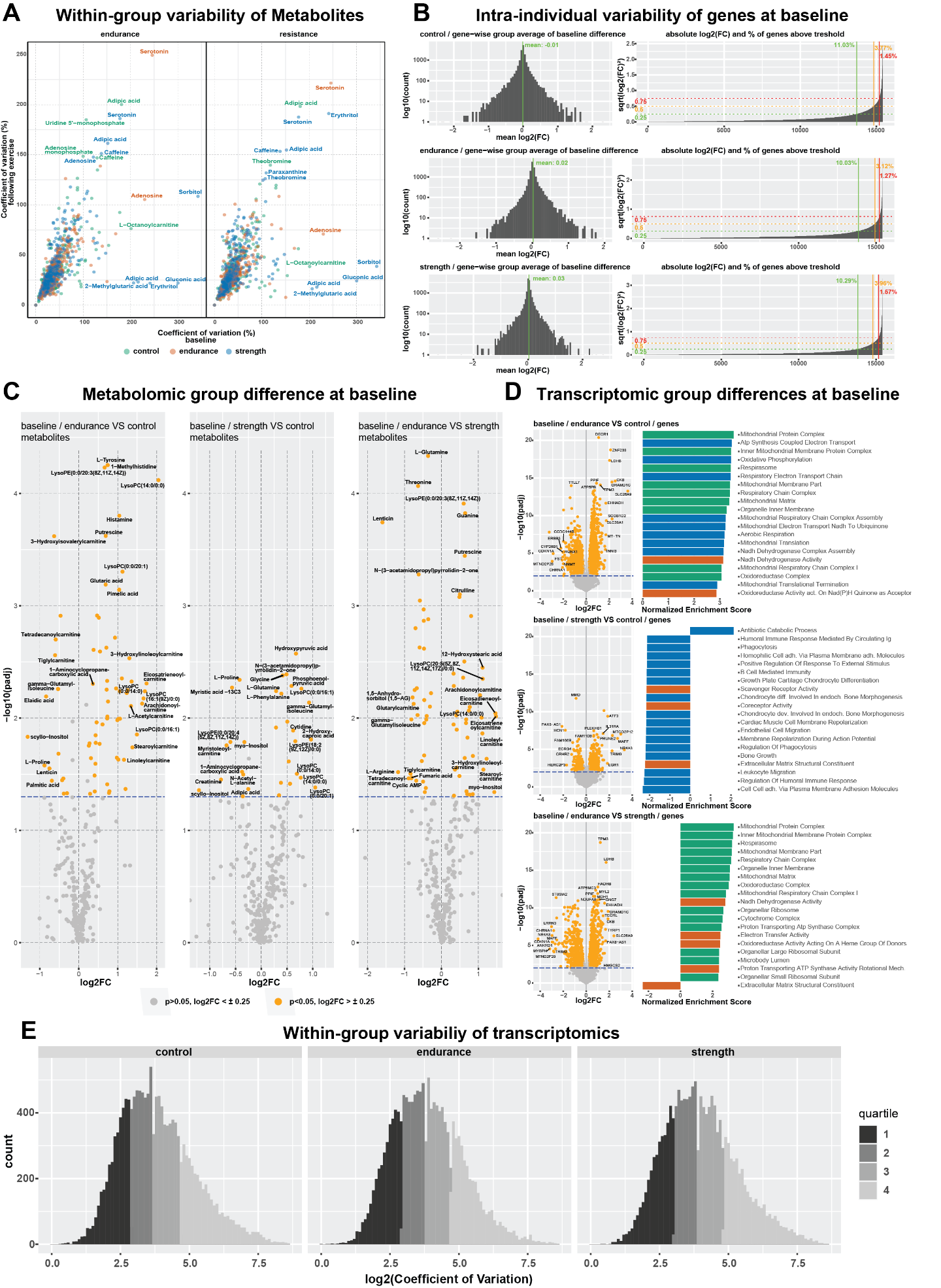


**Figure S2:** **Intra-individual and -group differences and variation on transcriptomic and metabolomic level**

(A) (A) Within subject group variation of metabolites at baseline (x-axis) and following acute exercise (mean of time course; y-axis) with the top metabolites in both dimensions annotated. (B) Intra-individual variability by gene-wise fold change analysis with mean and absolute fold change and proportion of genes above log fold change thresholds of 0.25, 0.5 and 0.75 separated by group. (C) Direct differential expression analysis of metabolites at baseline as volcano plot. (D) Direct differential gene expression analysis of groups at baseline as volcano plot and the top 20 pathways of each gene set based on normalized enrichment score from gene set enrichment analysis. (E) Density plot of the within-group coefficient of variation (CoV) for each gene separated by group. Quartiles of each set based on CoV are shown with different shades. CoV is presented as log2 transformation.


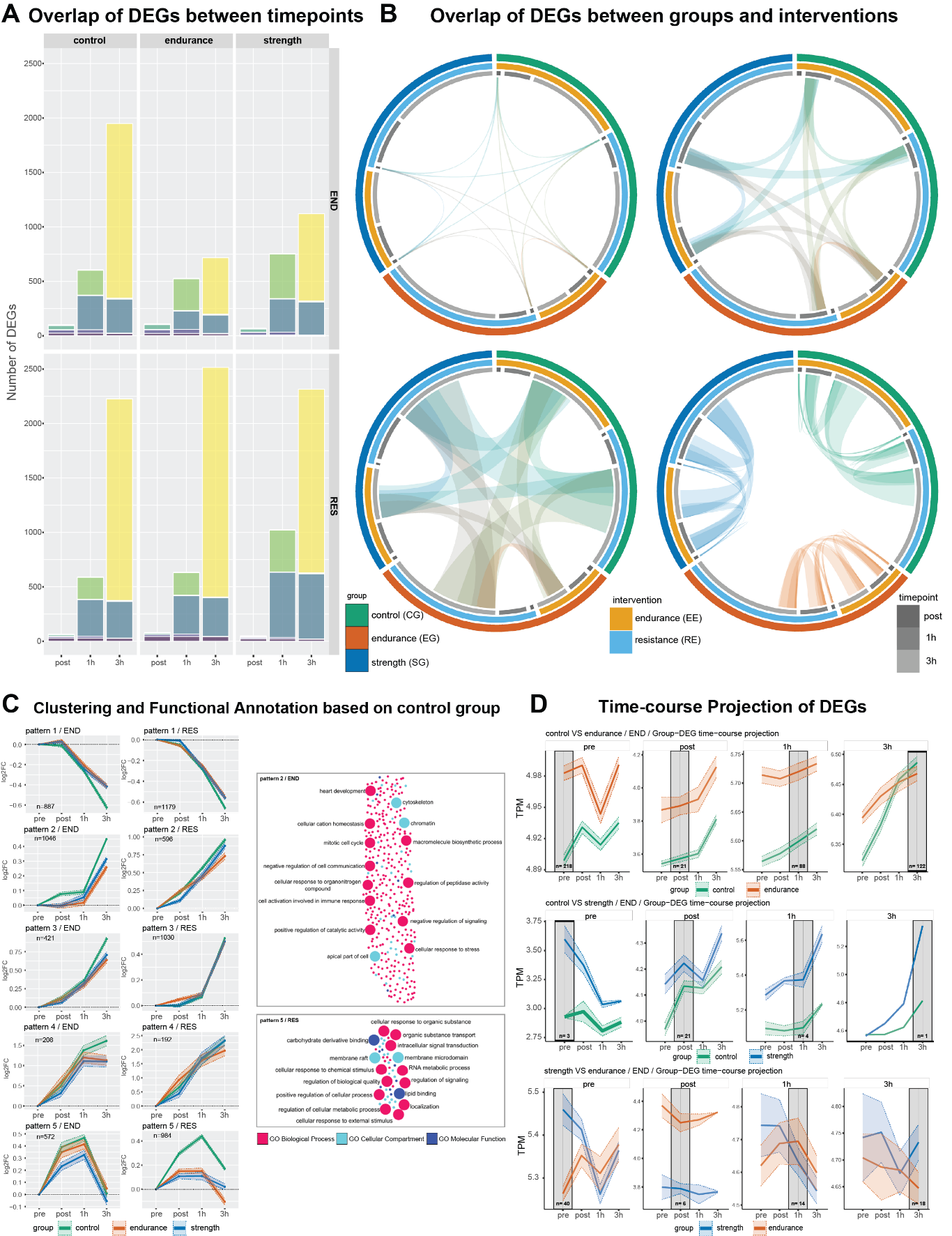


**Figure S3: Analysis of DEG overlaps and functional annotation of clusters**

(A) Overlaps of DEGs compared to pre-timepoint across the three post timepoints within control (CG), endurance (EG) and strength group (SG) in response to acute endurance (EE) and resistance exercise (RE). Colors represent sets of identical genes within each group-intervention pair. Genes common to all timepoints (core genes) ranged from 45 in EG performing RE (59% of all DEGs at post) to 6 in SG performing EE (9%). RE resulted in a higher proportion of core genes than EE across all groups. (B) Intra-group overlap of genes between groups and interventions at pre, post, 1h and 3h timepoints and of timepoints and acute interventions within groups. (C) Comparison of gene cluster trajectories based on unsupervised clustering of DEGs of the control group (CG) in response to both EE and RE. (D) Time-course projection (plotting of DEGs from one timepoint across the remaining timepoints) of genes significantly different between two groups at individual timepoints (solid line). Dotted lines show the confidence interval.


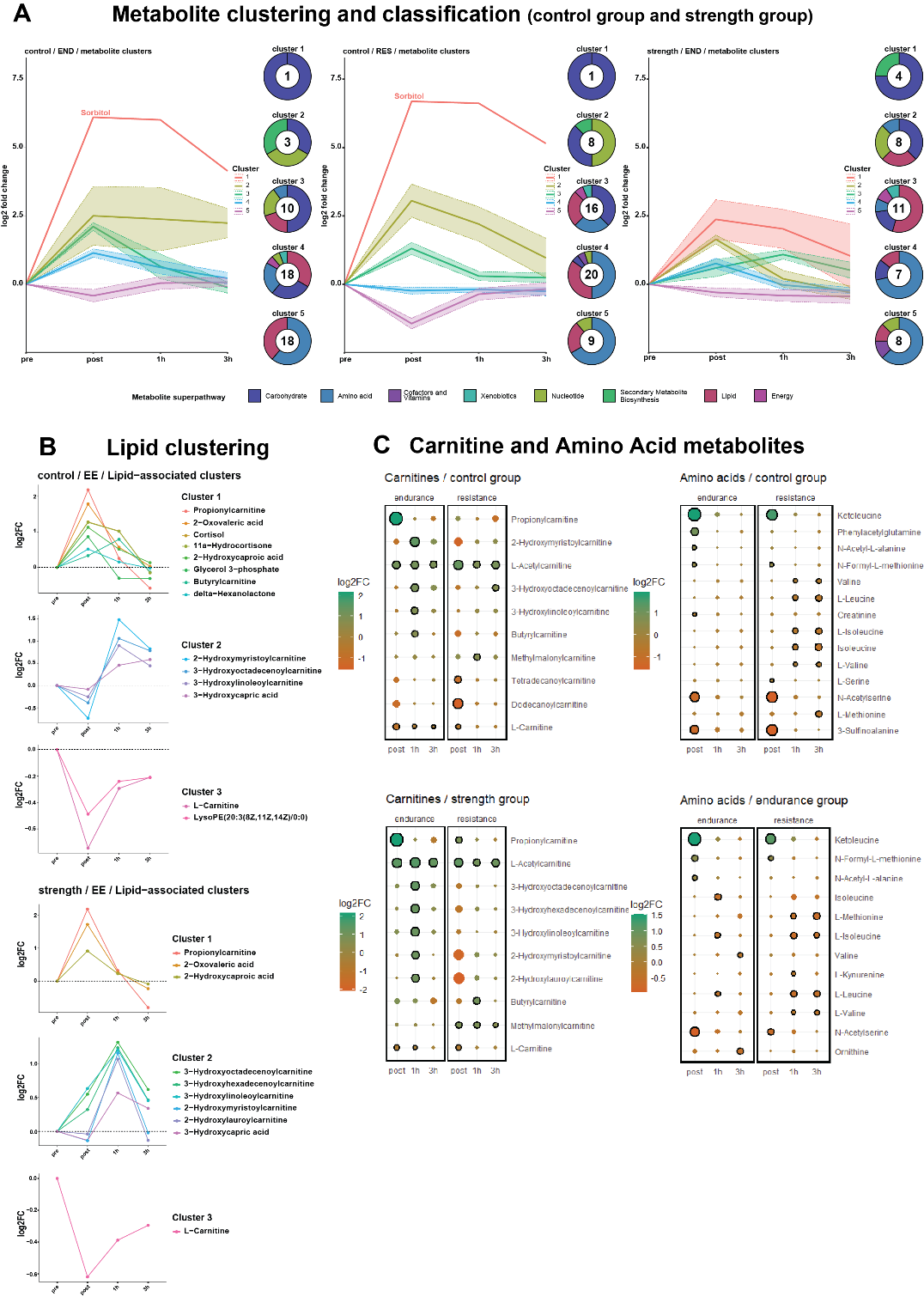


**Figure S4:** **Clustering and classification of metabolites**

*(A) Clustering and classification of metabolites of control (CG) and strength group (SG) in response to acute endurance (EE) and resistance exercise (RE). Solid lines show cluster mean, dotted lines show the confidence interval. Numbers inside the donuts represent the number of metabolites in the cluster. (B) Isolated clustering of all metabolites associated with lipids or lipid metabolism in control (CG) and strength group (SG) in response to acute endurance exercise. (C) Analysis of carnitines in control (CG) and strength group (SG) and amino acids in control (CG) and endurance group (EG) in response to both forms of acute exercise. Color and size of dots represent effect size and direction. Dots with solid black circles are statistically significant timepoints compared to pre timepoint.*


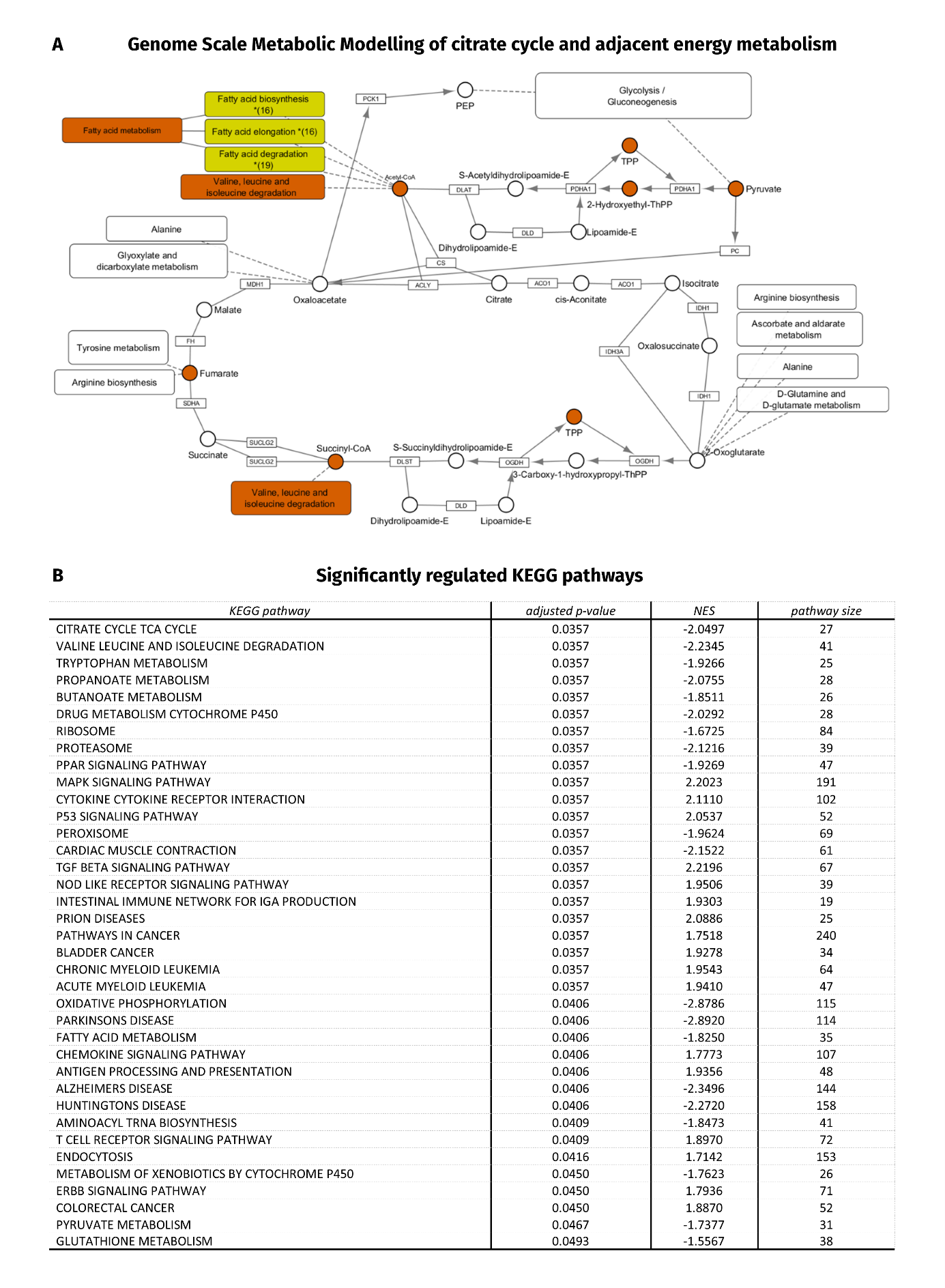


**Figure S5: Genome Scale Metabolic Modelling (GEM) and KEGG mapping**

*(A) KEGG mapping of the results of GEM analysis of endurance athletes (EG) performing acute endurance exercise (EE) comparing post timepoint with pre. Shown is the significantly affected citrate cycle pathway together with neighboring metabolic pathways. Orange color are reporter metabolites and pathways that are significantly downregulated. Yellow color are pathways in which a high number of their member reporter metabolites are downregulated (number of reporter metabolites in parenthesis). (B) Significantly regulated KEGG pathways in endurance trained athletes comparing post with pre timepoint immediately following acute endurance exercise.*


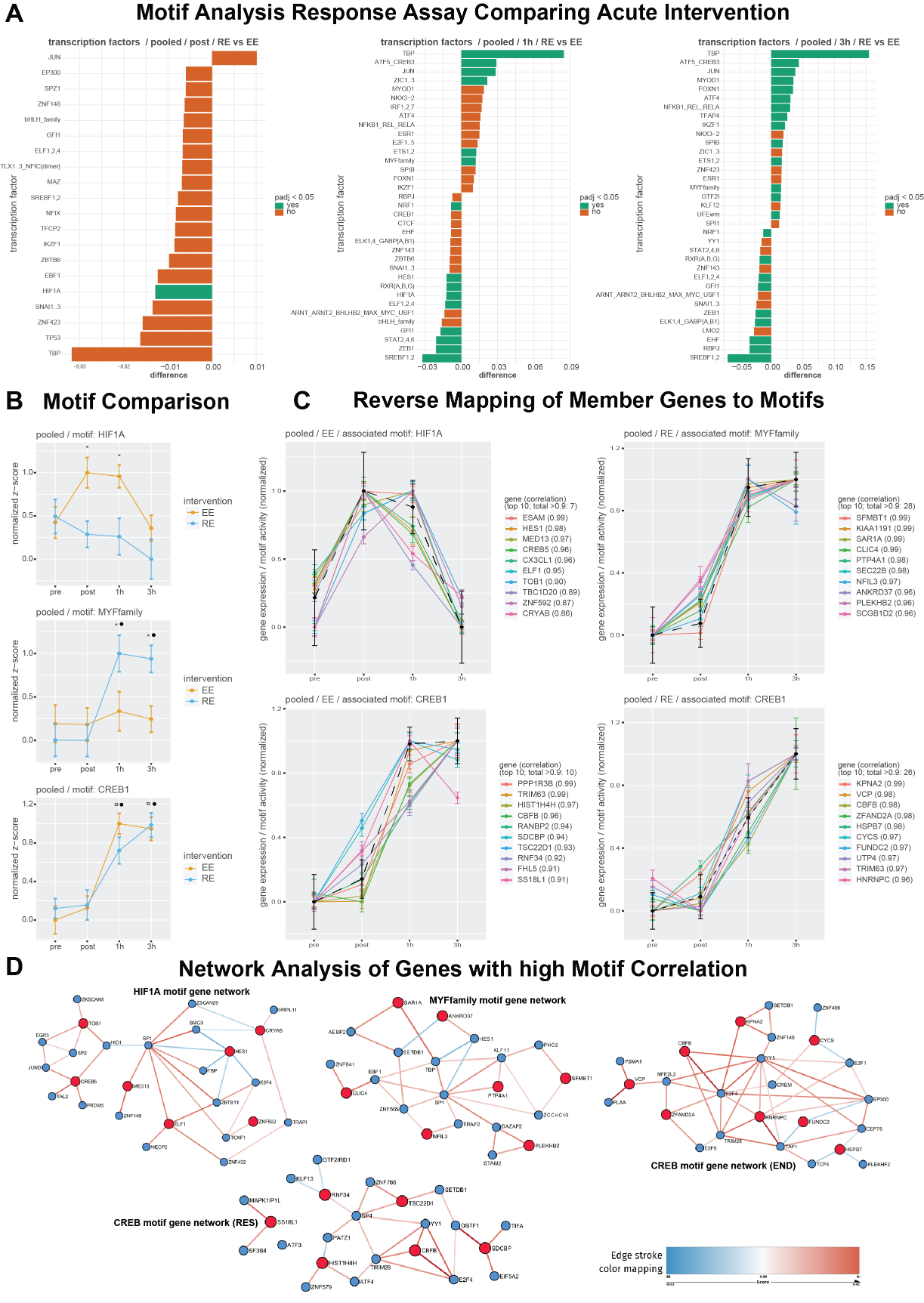


**Figure S6: Motif Activity Response Assay (MARA) and network analysis using TCSBN**

*(A) MARA comparing acute endurance (EE) and resistance exercise (RE) for all groups pooled at post, 1h and 3h timepoints (B) Direct comparison of motif activity time-course for the motifs HIF1A, MYFfamily and CREB. (C) Reverse mapping of motif member genes to motif activity (dashed line) using curve correlation. Top 10 genes are plotted, number of genes with correlation > 0.9 and individual gene correlation in parenthesis. (D) Network analysis of genes identified in reverse mapping (red dots) using the tissue and cancer specific biological networks (TCSBN) database. Edge colors represent positive (red) or negative (blue) connection of genes within the network.*
